# PINK1 protects against dendritic cell dysfunction during sepsis through the regulation of mitochondrial quality control

**DOI:** 10.1186/s10020-023-00618-5

**Published:** 2023-02-21

**Authors:** You Wu, Longwang Chen, Zhimin Qiu, Xijing Zhang, Guangju Zhao, Zhongqiu Lu

**Affiliations:** 1 Department of Emergency, First Affiliated Hospital of Wenzhou Medical University, Shangcai Road, Ouhai District, Wenzhou, 325000 Zhejiang China; 2 Department of Critical Care Medicine, Xijing Hospital, Xi’an, 710000 Shaanxi China

**Keywords:** Sepsis, Dendritic cells, PINK1, Mitophagy, Mitochondrial fission, Mitochondrial quality control

## Abstract

**Background:**

Dendritic cell (DC) dysfunction plays a central role in sepsis-induced immunosuppression. Recent research has indicated that collective mitochondrial fragmentation contributes to the dysfunction of immune cells observed during sepsis. PTEN-induced putative kinase 1 (PINK1) has been characterized as a guide for impaired mitochondria that can keep mitochondrial homeostasis. However, its role in the function of DCs during sepsis and the related mechanisms remain obscure. In our study, we elucidated the effect of PINK1 on DC function during sepsis and its underlying mechanism of action.

**Methods:**

Cecal ligation and puncture (CLP) surgery and lipopolysaccharide (LPS) treatment were used as in vivo and in vitro sepsis models, respectively.

**Results:**

We found that changes in mitochondrial PINK1 expression of DCs paralleled changes in DC function during sepsis. The ratio of DCs expressing MHC-II, CD86, and CD80, the mRNAs level of dendritic cells expressing TNF-α and IL-12, and the level of DC-mediated T-cell proliferation were all decreased, both in vivo and in vitro during sepsis*,* when *PINK1* was knocked out. This suggested that *PINK1* knockout prevented the function of DCs during sepsis. Furthermore, *PINK1* knockout inhibited Parkin RBR E3 ubiquitin protein (Parkin)-dependent mitophagy and enhanced dynamin-related protein 1 (Drp1)-related mitochondrial fission, and the negative effects of *PINK1* knockout on DC function following LPS treatment were reversed by Parkin activation and Drp1 inhibitor. Knockout of *PINK1* also increased apoptosis of DCs and the mortality of CLP mice.

**Conclusion:**

Our results indicated that PINK1 protected against DC dysfunction during sepsis through the regulation of mitochondrial quality control.

**Supplementary Information:**

The online version contains supplementary material available at 10.1186/s10020-023-00618-5.

## Introduction

Sepsis is a life-threatening organ dysfunction caused by a dysregulated host response to infection. Despite advances in the understanding and treatment of sepsis, high mortality and morbidity associated with sepsis continue to be a significant worldwide burden on society (Cecconi et al. 2018; Evans et al. 2021). Thus, a significant need remains to understand the pathophysiology of sepsis further and seek novel therapeutic strategies for treating sepsis.

It has been reported that poor outcomes of septic patients are associated with the occurrence of immunosuppression (Hotchkiss et al. 2013; Venet and Monneret 2018). Sepsis-associated immunosuppression is induced by aberrant immune responses and increased apoptosis of immune cells, including DCs, macrophages, and T lymphocytes. (Stolk et al. 2020). DCs are characterized as professional antigen-presenting cells (APCs) with the capability to activate naïve T cells and are a bridge between innate immunity and adaptive immunity. In response to stimuli, DCs mature into APCs and present major histocompatibility complex-II (MHC-II), CD80, and CD86 on their surface (Anderson et al. 2021; Waisman et al. 2017). During the progression of sepsis, the number of DCs is decreased, and DC function is impaired (Fan et al. 2015; Wang et al. 2015). It has been reported that improving DC function may alleviate immunosuppression and mortality during sepsis (Thwe et al. 2019). However, the potential mechanisms of DC dysfunction remain unclear during sepsis. Our previous research found that the imbalance of mitochondrial dynamics leads to increased mitochondrial fragmentation, which was responsible for T cell apoptosis under sepsis (Wu et al. 2019). Furthermore, recent studies have demonstrated that collective mitochondrial fragmentation is involved in the immune tolerance and apoptosis of immune cells (Mukherjee et al. 2022; Zheng et al. 2019). Therefore, we hypothesize that eliminating mitochondrial fragmentation and keeping mitochondrial homeostasis may improve DC function, which may mitigate immunosuppression.

PINK1 has been characterized to be a guide for impaired mitochondria. Previous research have shown that PINK1 deficiency contributes to the severity of organ dysfunction during sepsis (Tang et al. 2018). For example, PINK1 deficiency aggravates lipopolysaccharide (LPS)-induced lung injury and cardiac apoptosis and hypertrophy (Faridvand et al. 2021; Jiao et al. 2021). In addition, emerging evidence suggests that activation of PINK1 represents a beneficial role in immunoregulation (Kim et al. 2021; Lizama and Chu 2021). However, it has been unclear whether the expression of PINK1 is changed in DCs during sepsis and, if it changes, how PINK1 regulates DC function.

In healthy mitochondria, PINK1 inserts into the mitochondrial inner membrane (IMM) and is recognized and cleaved by a matrix processing peptidase (Nguyen et al. 2016). Subsequently, PINK1 is released back to the cytosol and degraded by the proteasome. However, the import of PINK1 to the IMM is blocked when it detects a damaged mitochondrion, resulting in the accumulation of PINK1 on the outer mitochondrial membrane (OMM) (Nguyen et al. 2016; Sekine and Youle 2018). PINK1, which is activated through auto-phosphorylation, can phosphorylate ubiquitin, a substrate of PINK1, which then induces the recruitment of Parkin, a E3 ubiquitin ligase, to the damaged mitochondria (Cummins and Götz 2017; Green et al. 2010; Nguyen et al. 2016). After that, Parkin is activated by phosphorylation, which binds to OMM proteins and autophagy adaptor proteins, including optineurin (OPTN) and nuclear dot protein 52 (NDP52), ultimately resulting in mitophagy (Ziegler et al. 2018). Additionally, recent research has shown that activated PINK1 regulates mitochondrial dynamics (McLelland et al. 2014; Truban et al. 2017). Mitophagy and mitochondrial dynamics are the processes of mitochondrial quality control, which play a central role in keeping mitochondrial homeostasis. Nevertheless, the role of PINK1 in mitochondrial quality control and its relationship with DC function is unclear. Therefore, in the current study, we used *PINK1* knockout mice to explore the protective effects of PINK1 on DC function through regulating mitochondrial quality control during sepsis.

## Materials and methods

### Animals

Wild-type (WT) and *PINK1*^*−/−*^ male C57BL/6 mice were generated from Jackson Laboratories. All animals were housed for at least one week prior to the start of the experiments and provided with rodent food and water ad libitum. The animal experiments were conducted in accordance with the National Institutes of Health Guidelines for the Care and Use of Laboratory Animals and were approved by the Laboratory Animal Ethics Committee of Wenzhou Medical University, Wenzhou, China.

### CLP procedure

The male C57BL/6 mice was anesthetized by 5% chloral hydrate (350 mg/kg). Two-thirds of the cecum was ligated and punctured once by 21 G needle. After closing of the abdomen, 0.9% sterile saline (1.0 mL) was subcutaneously injected to resuscitate. Same surgery but without cecum ligation or puncture was proceed in mice of the sham group. The mortality rate for WT mice within 5 days of CLP was approximately 50%.

### Isolation of splenic DCs and T lymphocytes

The spleens of septic mice were harvested and washed with phosphate-buffered saline (PBS). Mononuclear cells were separated from the splenic tissue using 40 μm meshes and mononuclear cells were obtained through density gradient centrifugation using NycoPrep (Axis-shield Co., Norway). Splenic DCs were obtained using CD11c MACS microbeads (Miltenyi Biotech, Bergisch Gladbach, Germany) while T lymphocytes were isolated using CD4 MACS microbeads (Miltenyi Biotech). Immature CD11c^+^ DCs were cultured in RPMI 1640 medium supplemented with 10% fetal calf serum (FCS) at 37 °C and 5% CO_2_. In vitro sepsis models, the CD11c^+^ DCs were treated with 1000 ng/mL LPS (*Escherichia coli* strain, Sigma).

### Flow cytometry analysis

CD11c^+^ DCs (3 × 10^5^) were suspended in 100 μL staining buffer of PBS and 0.1% sodium azide and then stained for 15 min at 4 °C with FITC-conjugated IgG specific for MHC-II (), PerCP-Cy5.5-conjugated IgG specific for CD80, or APC-conjugated IgG specific for CD86. The DCs were subsequently washed with PBS and fixed with 1% paraformaldehyde and analyzed using a FACScan system (BD Biosciences, Mountain View, CA, USA).

### Quantitative real-time reverse transcription PCR (RT-PCR)

The cellular mRNA was isloated from the cells using TRIzol reagent (Invitrogen, Carlsbad, CA, USA) and the concentration of the mRNA was determined spectrophotometrically at 260 nm. A 1 μg aliquot of each mRNA sample was used as template along with oligo dT and Superscript II reverse transcriptase to generate complementary DNA (cDNA). The cDNAs were then used for analysis to determine the relative expression of PINK1, LC3-II, Mfn2, Drp1, TNF-α and IL-12. The primers used are provided in Table 1. The thermal cycling conditions were set According to the manufacturer’s instructions, the conditions of thermal cycling were set. Relative expression levels of mRNA were measured using the ∆∆Ct method.

**Table 1:** Primer Sequence Table

Primer	
Pink1	
Forward	5′-GAGCAGACTCCCAGTTCTCG-3′
Reverse	5′-GTCCCACTCCACAAGGATGT-3′
LC3-II	
Forward	5′-CAAGCCTTCTTCCTCCTGGTGAA-3′
Reverse	5′-CCATTGCTGTCCCGAATGTCTCC-3′
Mfn2	
Forward	5′-GCATTCTTGTGGTCGGAGGAGTG-3′
Reverse	5′-TGGTCCAGGTCAGTCGCTCATAG-3′
Drp1	
Forward	5′-TGGTGAACCGGTGGATGATA-3′
Reverse	5′-CACCACCGCATAGCTCAGAA-3′
IL-12	
Forward	5′-CAGAAACCTCCTGTGGGAGA-3′
Reverse	5′-GGAGCTCAGATAGCCCATCA-3′
TNF-α	
Forward	5′-ACGGCATGGATCTCAAAGAC-3′
Reverse	5′-GTGGGTGAGGAGCACGTAGT-3′
β-actin	
Forward	5′-ATTGGCAATGAGCGGTTCCG-3′
Reverse	5′-AGGGCAGTGATCTCCTTCTG-3′

### T-cell proliferation assays

T cells were isolated and incubated with 5 μg/mL Con A for 16 h. The T cells were subsequently co-cultured with pre-treated DCs at a ratio of 1:200 for 3 d. In vivo DCs were isolated from *pink1*^*−/−*^ and WT mice after CLP surgery while in vitro DCs were prepared by isolating DCs from WT mice and then stimulating them with 1000 ng/mL LPS for 4 h. To evaluate cell proliferation, 10 μL CCK8 (Dojindo, Kumomoto, Japan) was added to the in vivo and in vitro DCs and mixed. The optical density of the CCK8-treated cells was measured using a Spectra MR microplate reader (Dynex, Richfield, MN, USA).

### Mitochondrial fractionation

Mitochondria were isolated by differential centrifugation. Mitochondrial extraction buffer consisting of 250 mM sucrose, 1 mM DTT, 10 mM KCl, 1 mM EDTA, 1 mM EGTA, 1.5 mM MgCl_2_, phenylmethylsulfonyl fluoride, and 20 mM HEPES pH 7.4 (Applygen Technologies Inc, Beijing, China) was added to the prepared cells. The homogenates of cell lysate were centrifuged at 800 × *g* for 10 min at 4 °C and the supernatant then centrifuged at 15,000 × *g* for 10 min at 4 °C. After centrifugation, the supernatant represented the cytoplasmic fraction and the pellet contained the mitochondria. The pellet was re-suspended in mitochondrial lysis buffer (Beyotime Biotechnology, Shanghai, China) to obtain the mitochondrial fractions. Cytoplasmic and mitochondrial fractions (20 μg) were subjected to western blot analysis.

### Western blot analysis

The 20 μg aliquots of cytoplasmic and mitochondrial fractions were mixed with SDS-loading buffer and boiled at 95 °C for 3–5 min. Equal amounts of proteins were loaded and electrophoretically separated using SDS-PAGE (Pulilai Co, Beijing, China). The separated proteins were transferred to nitrocellulose membranes and the membranes then incubated at 4 °C overnight with the following primary antibodies: anti-PINK1 (1:1000, ab23707, Abcam, Boston, MA, USA), anti-parkin (1:1000, ab77924, Abcam), anti-LC3B (1:1000, #3868, Cell Signaling Technology, Danvers, MA, USA), anti-P62 (1:1000, #39749, Cell Signaling Technology), anti-Tomm20 (1:1000, #42406, Cell Signaling Technology), anti-COX IV (1:1000, ab33985, Abcam), anti-Drp1 (1:1000, #5391, Cell Signaling Technology) anti-Mfn2 (1:1000, #9482, Cell Signaling Technology), anti-OPA1 (1:1000, #80471, Cell Signaling Technology), anti-cleaved caspase-3 (1:1000, #9661, Cell Signaling Technology) or anti-β-actin (1:5000, ab8226, Abcam). The membranes were then incubated with the appropriate secondary antibodies for 1 h at room temperature. The protein bands were visualized using enhanced chemiluminescence (Amersham Bioscience, Uppsala, Sweden) and quantified using Image J software (NIH).

### Laser scanning confocal microscopy (LSCM)

After the various treatments, the DCs were fixed with 4% paraformaldehyde for 20 min at room temperature. To evaluate mitophagy, the fixed DCs were incubated with Mitophagy-Tracker Yellow (Dojindo) for 20–30 min in darkness at room temperature, washed three times with PBS, and then incubated with Lyso-Tracker Green (Dojindo) for 20–30 min in darkness. After being washed with PBS, the DCs were plated onto glass slides, and stained with 4′,6-diamidino-2-phenylindole (DAPI). A laser scanning confocal microscope (Leica, Wetzlar, Germany) was used to observe the cells. To evaluate mitochondrial morphology, fixed DCs were incubated with the dapoxyl probe Mito-Tracker Red (Dojindo) for 20–30 min in darkness at 4 °C. After being washed three times with PBS, the DCs were plated onto glass slides and stained with DAPI. The Leica laser scanning confocal microscope (Leica, Mannheim, Germany) was used to observe the cells (Additional file 3).

### Transmission electron microscopy

Purified DCs (4 × 10^6^ cells/mL) were fixed in 2.5% glutaraldehyde. The ultrastructure of the DCs was photographed under a transmission electron microscope (Zeiss, Oberkochen, Germany).

### Parkin RNA lentivirus generation and transfection

Over-expression RNA to Parkin (Parkin-LV-RNA) was synthesized by Shanghai GeneChem Co, Ltd (Shanghai, China). Transfection was performed according to the manufacture’s instruction. The transduction efficiency for dendritic cells in vitro was > 80%. After transfection for 72 h, the transduction efficiency was checked by Western blotting.

### 5-bromo-2′-deoxyuridine (BrdU) staining

T cells were isolated and permeabilized in 0.1% Triton X-100 for 20 min. Then the T cells were incubated in 2N HCl for 30 min at 37 °C, washed three times for 10 min with TBST. The T cells were blocked for 1.5 h in TBS and incubated with the primary antibody (anti-BrdU antibody, 1:500, Abcam, ab6326) at 4 °C overnight. After being washed three times for 10 min with TBST, the T cells were then incubated with the appropriate secondary antibodies for 1.5 h at room temperature. After being washed three times for 10 min with TBST, the T cells were mounted and imaged on fluorescence microscopy.

### Statistical analysis

Data were represented as the mean ± standard deviation (SD) of more than three independent experiments. Multiple groups were analyzed using one-way analysis of variance (ANOVA) or two-way ANOVA. Two groups comparisons were analyzed using unpaired Student’s *t*-test, as required after testing for normality. The survival rate was carried out with Kaplan–Meier survival curves and long-rank test. Statistical significance was accepted at *P* < 0.05. GraphPad Prism 6 (San Diego, CA, USA) was used for statistical analysis and figure preparation.

## Results

### Change in DC function after CLP

**Fig. 1:**
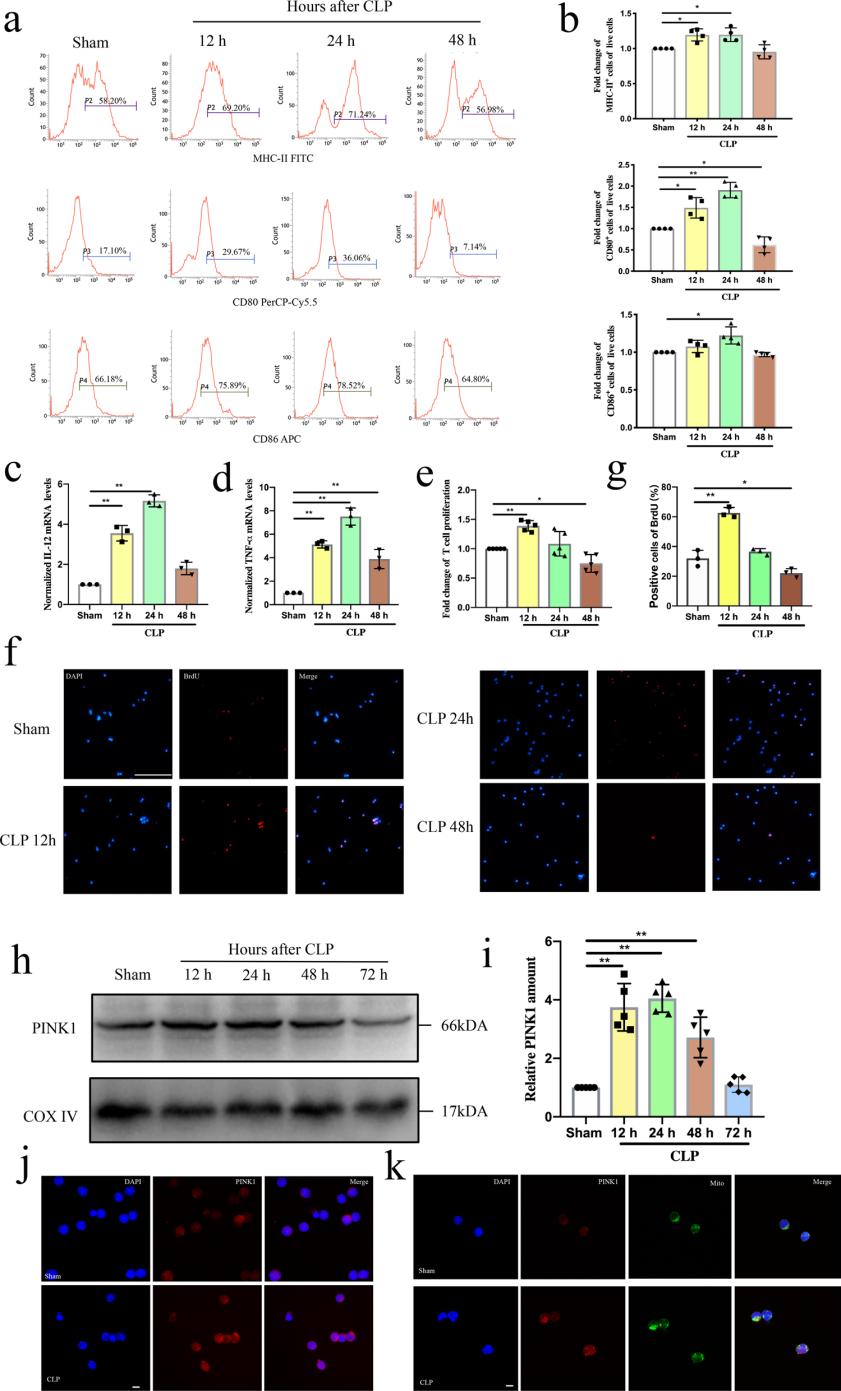
Change in DC function and mitochondrial PINK1 expression of DCs at different time after CLP. **a, b** Representative flow cytometric analysis of MHC-II, CD80, and CD86 expression and statistical analysis of relative MHC-II, CD80, and CD86 expression on DCs in each group (n = 4). **c** IL-12 mRNA expression was analyzed by PCR and statistical analysis of relative mRNA expression of IL-12 on DCs in each group (n = 3). **d** TNF-α mRNA expression was analyzed by PCR and statistical analysis of relative mRNA expression of TNF-α on DCs in each group (n = 3). **e** CD4^+^T cells were activated with ConA (5 μg/mL) for 18 h, and then were co-cultured with DCs at a ratio of 1:100. CCK-8 was used to test T cell proliferation. Representative expression of T cell proliferation in each group (n = 5). **f, g** Representative images of BrdU staining (red) and DAPI (blue) on DCs in sham and CLP group and statistical analysis of positive cells (n = 3). **h, i** Representative western blots of PINK1 and COXIV in each group and statistical analysis of relative PINK1 expression (n = 5). **j** Representative fluorescence images of PINK1 expression on DCs in sham and CLP group, including PINK1 protein (red) and nucleus (blue). **k** Representative fluorescence images of the co-localization of PINK1 and mitochondria on DCs in sham and CLP group, including PINK1 protein (red), mitochondria (green) and nucleus (blue). Results of experiments were shown as the mean ± SD. Statistical significance was assessed using one-way ANOVA analysis with Dunnett’s multiple comparisons test. *P* values are reported as follows: * < 0.05; ** < 0.01

The changes in DC function may contribute to immunosuppression and mortality during sepsis (Yao et al. 2022). To verify this process, we first examined the changes in DC function during sepsis in vivo. Mice were subjected to CLP to induce experimental sepsis. Splenic DCs were then obtained at different times after CLP and evaluated for expression of MHC-II, CD86, and CD80, which are phenotypic markers of DC maturation. Flow cytometry analysis showed that the ratio of DCs expressing MHC-II, CD86, and CD80 was increased in the CLP mice compared to that of the sham group, peaked at 24 h post CLP (Fig. 1a, b, *P* < 0.01 or *P* < 0.05). Furthermore, mRNA expression levels of cytokines reflecting DC function, including tumor necrosis factor (TNF)-α and interleukin (IL)-12, were increased in the CLP mice, peaked at 24 h after CLP, suggesting that DC function was impaired after 48 h of CLP (Fig. 1c, d, *P* < 0.01). The ability of DCs to induce the CD4^+^ T cells proliferation was evaluated to further verify that DC function. As shown in Fig. 1e–g, both CCK8 and BrdU staining results showed that the proliferation of CD4^+^ T cells was increased at 12 h post CLP and decreased at 48 h post CLP (*P* < 0.01 or *P* < 0.05). Consistent with the previous study, DC function is impaired in the late stage of sepsis (Luan et al. 2019).

### Change in mitochondrial PINK1 expression of DCs after CLP

We then determined the expression of mitochondrial PINK1 in DCs at different times after CLP. In the qPCR assay, mRNA expression levels of PINK1 were increased in CLP mice, peaked at 24 h after CLP (Additional file 1: Fig. S1a, *P* < 0.01). To further determine the expression of mitochondrial PINK1 in DCs, we isolated mitochondria from DCs and analyzed quantitation based on western blot assay. As shown in Fig. 1h, i, the expression of mitochondrial PINK1 in DCs was increased in the CLP mice compared to that in sham mice, peaked at 24 h after CLP (*P* < 0.01). Fluorescence images revealed that PINK1 expression and the interaction of PINK1 and mitochondria, as shown by co-localization of PINK1 and mitochondria in the fluorescence images, were increased at 24 h after CLP (Fig. 1j, k). Together, these results demonstrate that the change in mitochondrial PINK1 expression of DCs paralleled the changes in DC function after CLP.

### Change in DC function following LPS treatment

**Fig. 2:**
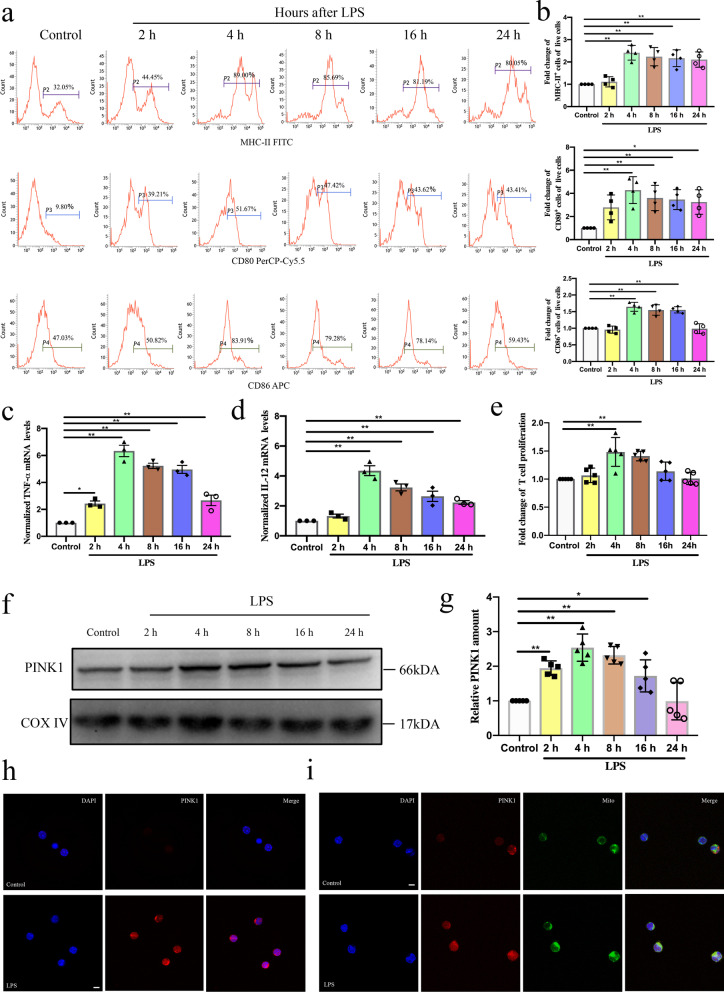
Change in DC function and mitochondrial PINK1 expression of DCs at different time following LPS treatment. **a, b** Representative flow cytometric analysis of MHC-II, CD80, and CD86 expression and statistical analysis of relative MHC-II, CD80, and CD86 expression on DCs in each group (n = 4). **c** TNF-α mRNA expression was analyzed by PCR and statistical analysis of relative mRNA expression of TNF-α on DCs in each group (n = 3). **d** IL-12 mRNA expression was analyzed by PCR and statistical analysis of relative mRNA expression of IL-12 on DCs in each group (n = 3). **e** CD4^+^T cells were activated with ConA (5 μg/mL) for 18 h, and then were co-cultured with DCs at a ratio of 1:100. CCK-8 was used to test T cell proliferation. Representative expression of T cell proliferation in each group (n = 5). **f, g** Representative western blots of PINK1 and COXIV in each group and statistical analysis of relative PINK1 expression (n = 5). **h** Representative fluorescence images of PINK1 expression on DCs in Control and LPS group, including PINK1 protein (red) and nucleus (blue). **i** Representative fluorescence images of the co-localization of PINK1 and mitochondria on DCs in Control and LPS group, including PINK1 protein (red), mitochondria (green) and nucleus (blue). Results of experiments were shown as the mean ± SD. Statistical significance was assessed using one-way ANOVA analysis with Dunnett’s multiple comparisons test. *P* values are reported as follows: * < 0.05 and ** < 0.01

Next, we examined the change in DC function during sepsis in vitro. DCs were exposed to LPS to mimic sepsis. Flow cytometry analysis revealed that the ratio of DCs expressing MHC-II, CD86, and CD80 was increased following LPS treatment, peaked at 4 h, while declined after 8 h (Fig. 2a, b, *P* < 0.01 or *P* < 0.05). Consistent with phenotypic maturation of DCs, the mRNA expression levels of cytokines TNF-α and IL-12 were increased following LPS treatment, peaked at 4 h, while declined after 8 h, suggesting that DC function was impaired at 8 h post LPS treatment (Fig. 2c, d, *P* < 0.01 or *P* < 0.05). Furthermore, as shown in Fig. 2e, the proliferation of CD4^+^ T cells that were co-cultured with DCs was increased at 4 h and 8 h following LPS treatment (*P* < 0.01).

### Change in mitochondrial PINK1 expression of DCs following LPS treatment

To determine changes in mitochondrial PINK1 expression during sepsis in vitro, the levels of mitochondrial PINK1 expression in DCs were evaluated at different times following LPS treatment. In the qPCR assay, mRNA expression levels of PINK1 were increased following LPS treatment, peaked at 4 h, while declined after 8 h (Additional file 1: Fig. S1b, *P* < 0.01). In addition, as shown in Fig. 2f, g, mitochondrial PINK1 expression levels in DCs were increased following LPS treatment, peaked at 4 h, while declined after 8 h (*P* < 0.01 or *P* < 0.05). Fluorescence imaging also showed that PINK1 expression was increased (Fig. 2h). Furthermore, the interaction of PINK1 and mitochondria was increased at 4 h following LPS treatment, as indicated by the co-localization of PINK1 and mitochondria in the fluorescence images (Fig. 2i). Collectively, the above noted results suggest that changes in DC function may be related to changes in mitochondrial PINK1 expression during sepsis.

### Knockout of *PINK1* prevented DC function following LPS treatment

**Fig. 3:**
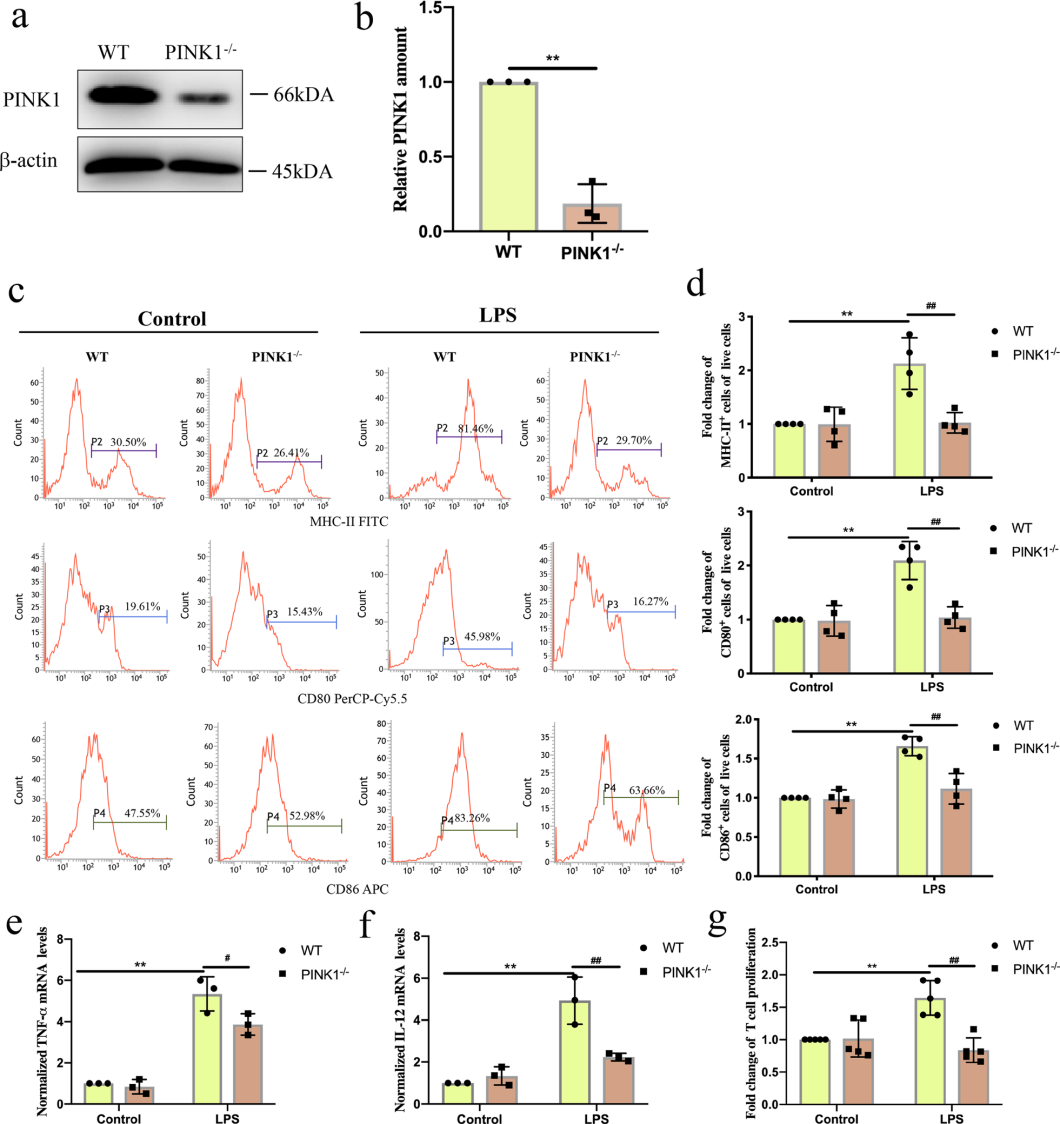
Knockout of *PINK1* prevented DC function following LPS treatment. Immature DCs from WT mice and *pink1* Knockout mice were cultured by PBS or LPS (1000 ng/mL) for 4 h. **a, b** Representative western blots of PINK1 and COXIV in each group and statistical analysis of relative PINK1 expression (n = 3). **c, d** Representative flow cytometric analysis of MHC-II, CD80, and CD86 expression and statistical analysis of relative MHC-II, CD80, and CD86 expression on DCs in each group (n = 4). **e** TNF-α mRNA expression was analyzed by PCR and statistical analysis of relative mRNA expression of TNF-α on DCs in each group (n = 3). **f** IL-12 mRNA expression was analyzed by PCR and statistical analysis of relative mRNA expression of IL-12 on DCs in each group (n = 3). **g** CD4^+^ T cells were activated with ConA (5 μg/mL) for 18 h, and then were co-cultured with DCs at a ratio of 1:100. CCK-8 was used to test T cell proliferation (n = 5). Results of experiments were shown as the mean ± SD. Statistical significance was assessed using Student’s *t* test (b) and two-way ANOVA analysis with Sidak’s multiple comparisons test (**d**–**g**). *P* values are reported as follows: ^#^ < 0.05 and **, ^##^ < 0.01

To examine whether PINK1 affected DC function, we evaluated the phenotypic maturation and cytokine expression of DCs isolated from WT and *PINK1*^*−/−*^ mice following LPS treatment. As shown in Fig. 3a, b, PINK1 levels were lower in DCs isolated from *PINK1*^*−/−*^ mice compared with those isolated from WT mice (*P* < 0.01). Furthermore, knockout of *PINK1* reduced the ratio of DCs expressing MHC-II, CD80, and CD86 at 4 h post LPS treatment (Fig. 3c, d, *P* < 0.01). Knockout of *PINK1* in DCs also reduced the mRNA expression of TNF-α and IL-12 at 4 h following LPS treatment (Fig. 3e, f, *P* < 0.01 or *P* < 0.05). We further determined the effect of *PINK1* knockout on the ability of DCs to induce CD4^+^ T-cell proliferation following LPS treatment. As shown in Fig. 3g, knockout of *PINK1* reduced the LPS-induced increase in DC-mediated T-cell proliferation (*P* < 0.05). These findings demonstrate that knockout of *PINK1* prevented DC function following LPS treatment; however, the underlying mechanism requires further study.

### Mitophagy induction in DCs following LPS treatment

**Fig. 4:**
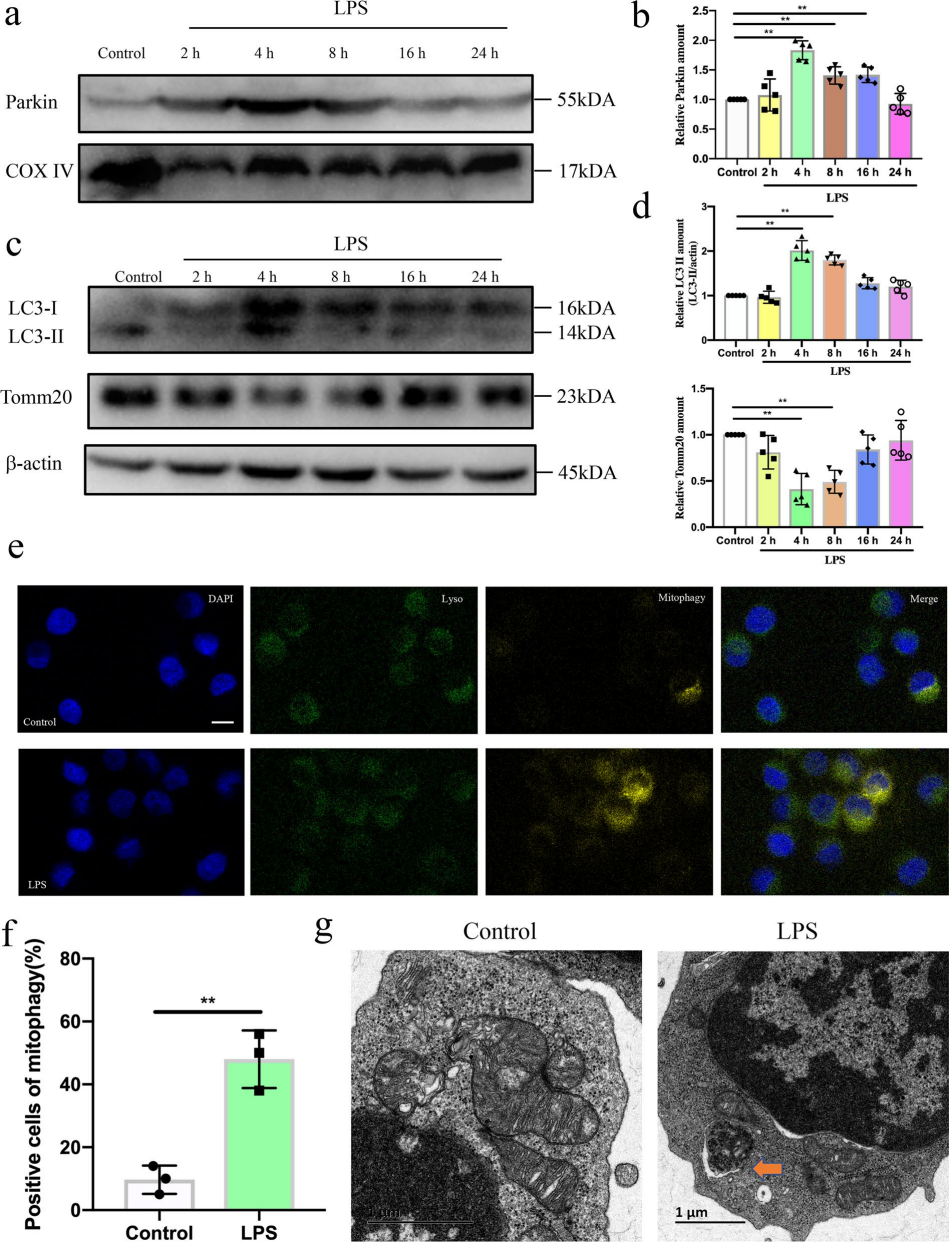
Mitohpgay induction in DCs at different time following LPS treatment. **a, b** Representative western blots of Parkin and COXIV in each group and statistical analysis of relative expression (n = 5). **c, d** Representative western blots of LC3, Tomm20 and β-actin in each group and statistical analysis of relative LC3II and Tomm20 expression (n = 5). **e, f** Representative fluorescence images of the co-localization of lysosome and mitophagy and statistical analysis of percentage of mitophagy-positive cells on DCs in sham and CLP group, including lysosome (green), mitophagy (yellow) and nucleus (blue) (n = 3). **g** Representative electron micrographs of mitophagosomes on DCs in Control and LPS group. Results of experiments were shown as the mean ± SD. Statistical significance was assessed using Student’s *t* test (**f**) and one-way ANOVA analysis with Dunnett’s multiple comparisons test (**b**, **d**). *P* values are reported as follows: ** < 0.01

Previous studies have demonstrated that PINK1-Parkin-dependent mitophagy represents a beneficial role in immunoregulation after sepsis (Gonzalez et al. 2014; Oami et al. 2017; Tran et al. 2011). Therefore, we investigated the occurrence of mitophagy in DCs following LPS treatment. The above results showed that mitochondrial PINK1 expression levels in DCs were increased following LPS treatment, peaked at 4 h, while declined after 8 h. PINK1 can phosphorylate ubiquitin, a substrate of PINK1, which then induces the recruitment of Parkin to the damaged mitochondria, ultimately resulting in mitophagy (Xu et al. 2020). We then determined the expression of mitochondrial Parkin in DCs at different times following LPS treatment. Consistent with changes in mitochondrial PINK1 expression, mitochondrial Parkin expression levels in DCs were increased following LPS treatment, peaked at 4 h (Fig. 4a, b, *P* < 0.01). Subsequently, we evaluated the expression levels of LC3, a marker for autophagy, and western blot analysis revealed that both mRNA and protein levels of LC3 II were increased following LPS treatment and peaked at 4 h, while declined after 8 h (Additional file 1: Fig. S1c, 4c, d, *P* < 0.01). Whereas the expression of OMM protein Tomm20 was reduced following LPS treatment and reached the lowest level at 4 h **(**Fig. 4c, d, *P* < 0.01 or *P* < 0.05), indicating that mitophagy was induced. To further prove the induction of mitophagy following LPS treatment, fluorescence was monitored to evaluate the delivery of mitochondria to lysosome. Mitochondrial fragments presented as distinct yellow puncta when they interacted with the lysosome. As shown in Fig. 4e, f, distinct yellow mitochondrial fragments were present in LPS-treated cells while no color staining was observed in the control cells (*P* < 0.01). We also examined mitophagy in DCs based on morphological changes detected using electron microscopy. The transmission electron microscopy results showed the presence of a mitophagosome in DCs following LPS treatment (Fig. 4g). Collectively, these results suggest that PINK1-Parkin-dependent mitophagy is induced during sepsis.

### Knockout of *PINK1* inhibited LPS-induced mitophagy in DCs and Parkin reversed the role of *PINK1* knockout on DC function

**Fig. 5:**
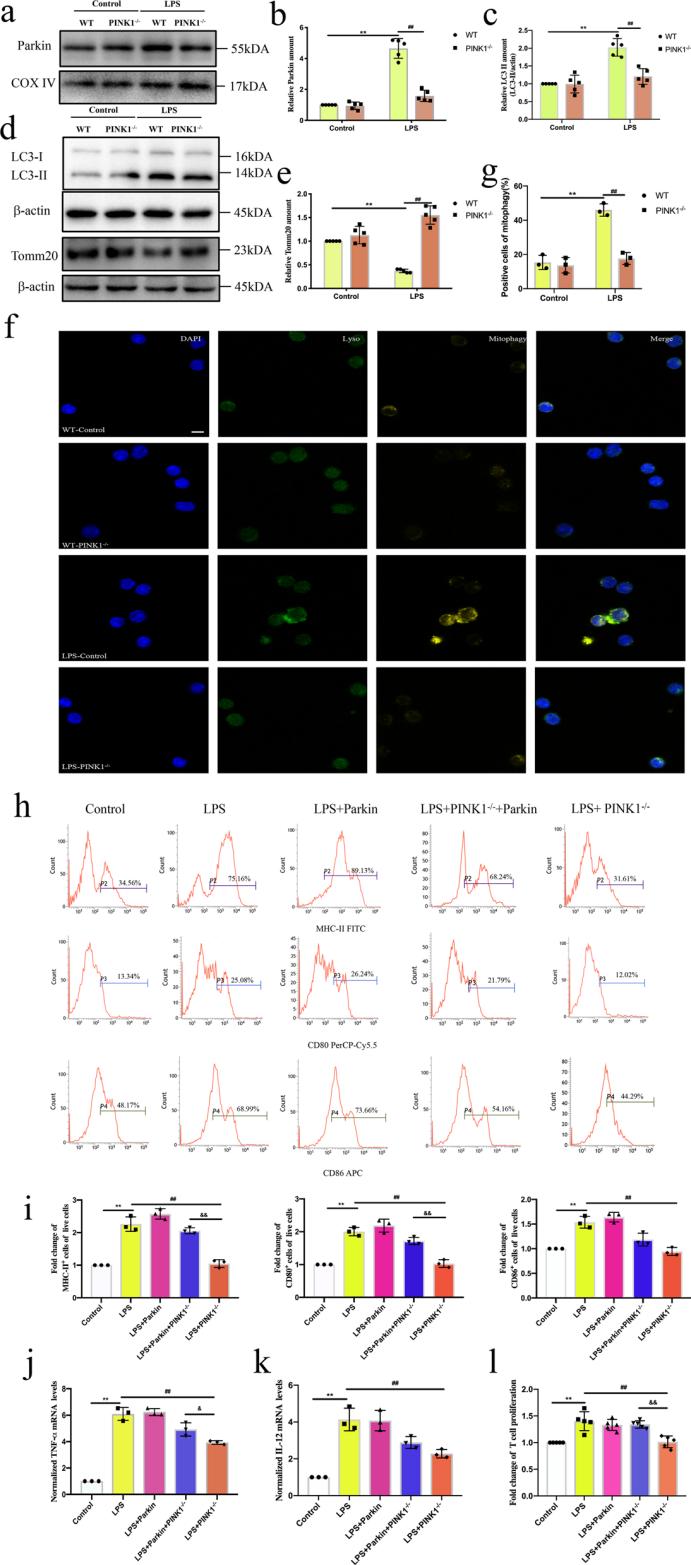
Knockout of *PINK1* inhibited LPS-induced mitophagy in DCs and Parkin reversed the role of *PINK1* knockout on DC function. Immature DCs from WT mice and *PINK1* knockout mice were cultured by PBS or LPS (1000 ng/mL) for 4 h. **a, b** Representative western blots of PARK2 and COXIV in each group and statistical analysis of relative PARK2 expression (n = 5). **c–e** Representative western blots of LC3, Tomm20 and β-actin in each group and statistical analysis of relative LC3II and Tomm20 expression (n = 5). **f, g** Representative fluorescence images of the co-localization of lysosome and mitophagy and statistical analysis of percentage of mitophagy-positive cells on DCs in each group, including lysosome (green), mitophagy (yellow) and nucleus (blue) (n = 3). **h, i** Representative flow cytometric analysis of MHC-II, CD80, and CD86 expression and statistical analysis of relative MHC-II, CD80, and CD86 expression on DCs in each group (n = 3). **j** TNF-α mRNA expression was analyzed by PCR and statistical analysis of relative mRNA expression of TNF-α on DCs in each group (n = 3). **k** IL-12 mRNA expression was analyzed by PCR and statistical analysis of relative mRNA expression of IL-12 on DCs in each group (n = 3). **l** Representative expression of T cell proliferation in each group (n = 5). Results of experiments were shown as the mean ± SD. Statistical significance was assessed using one-way ANOVA analysis with Sidak’s multiple comparisons test **i**–**l** and two-way ANOVA analysis with Sidak’s multiple comparisons test (**b**, **c**, **e**, **g**). *P* values are reported as follows: ^&^ < 0.05 and **, ^##^, ^&&^ < 0.01

To determine the role of PINK1 in LPS-induced mitochondrial quality control, we firstly evaluated the induction of Parkin-dependent mitophagy in DCs isolated from WT and *PINK1*^*−/−*^ mice following LPS treatment. As shown in Fig. 5a, b, knockout of *PINK1* reduced the expression levels of Parkin at 4 h following LPS treatment (*P* < 0.01). Compared with the Wild Type-LPS group, both mRNA and protein expression levels of LC3 II were down-regulated and the expression levels of Tomm20 were upregulated following LPS treatment in *PINK1*^*−/−*^-LPS group (Additional file 1: Fig. S1d, 5c–e, *P* < 0.01). In addition, knockout of *PINK1* reduced the amount of distinct yellow puncta of mitochondrial fragments in DCs following LPS treatment (Fig. 5f, g, *P* < 0.01), indicating that *PINK1* knockout inhibited LPS-induced Parkin-dependent mitophagy in DCs. Then, we determined whether the protective role of PINK1 on DC function was through Parkin. As shown in Additional file 2: Fig. S2a, b, overexpression of Parkin increased expression levels of LC3 II and reduced expression levels of Tomm20 in *PINK1*^*−/−*^ mice, indicating that Parkin recovered mitophagy that is inhibited by *PINK1* knockout (*P* < 0.01 or* P* < 0.05). Furthermore, Parkin reversed the negative role of *PINK1* knockout on DC function at 4 h following LPS treatment (Fig. 5h–l, *P* < 0.01 or *P* < 0.05).

### Knockout of *PINK1* inhibited LPS-induced mitochondrial dynamics and Mdivi-1 reversed the role of *PINK1* knockout on DC function

**Fig. 6:**
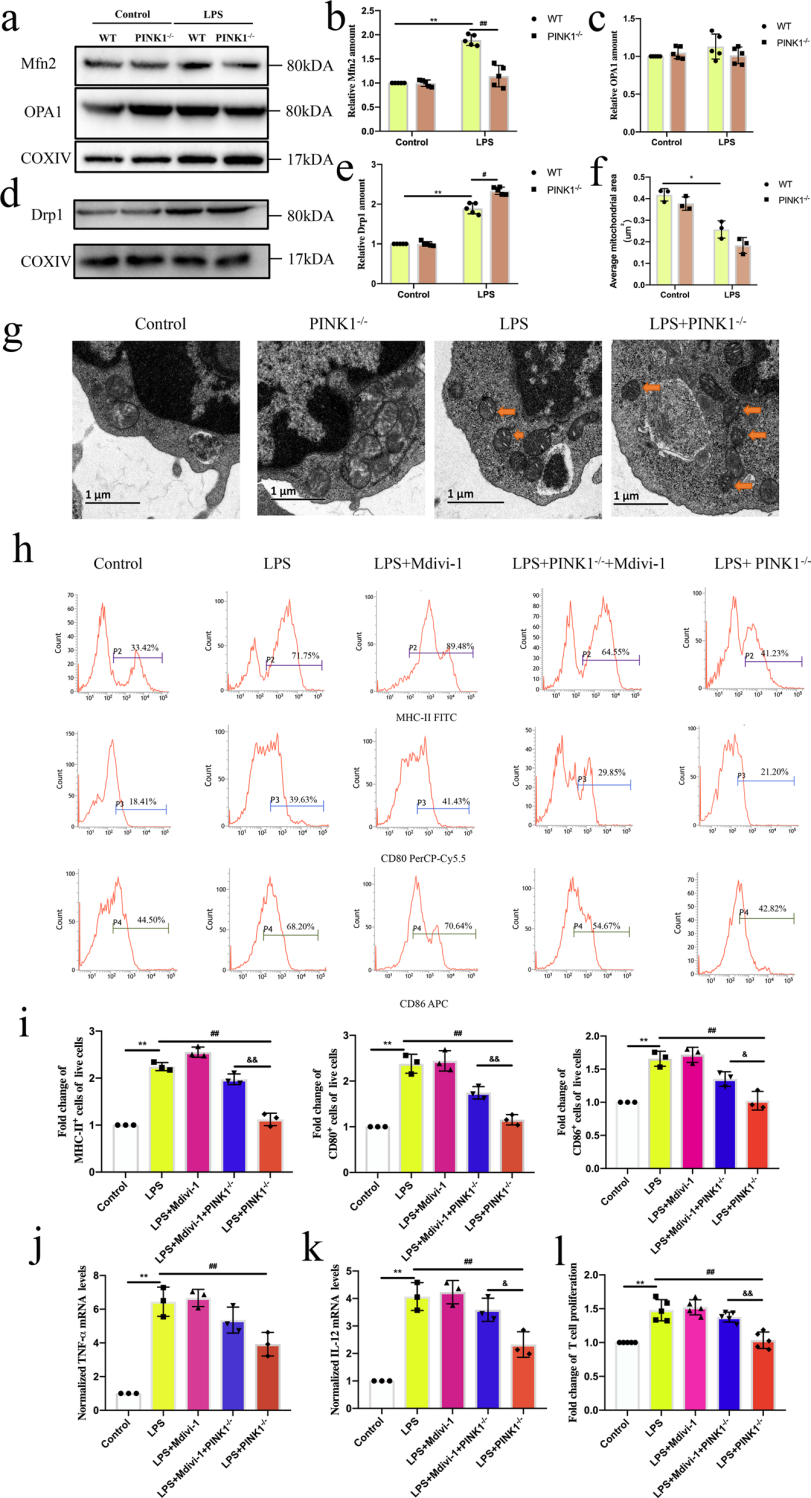
Knockout of *PINK1* inhibited LPS-induced mitochondrial dynamics and Mdivi-1 reversed the role of *PINK1* knockout on DC function**.** Immature DCs from WT mice were cultured by LPS (1000 ng/mL) and immature DCs from *PINK1* knockout mice were cultured by LPS or LPS + Mdivi-1 (20 μM) for 4 h. **a–c** Representative western blots of Mfn2, OPA1 and COXIV in each group and statistical analysis of relative Mfn2 and OPA1 expression (n = 5). **d, e** Representative western blots of Drp1 and COXIV in each group and statistical analysis of relative Drp1 expression (n = 5). **f, g** Representative electron micrographs of mitochondria on DCs in Control, PINK1^−/−^, LPS, and LPS + PINK1^−/−^ group and statistical analysis of average mitochondrial area (n = 3). **h, i** Representative flow cytometric analysis of MHC-II, CD80, and CD86 expression and statistical analysis of relative MHC-II, CD80, and CD86 expression on DCs in each group (n = 3). **j** TNF-α mRNA expression was analyzed by PCR and statistical analysis of relative mRNA expression of TNF-α on DCs in each group (n = 3). **k** IL-12 mRNA expression was analyzed by PCR and statistical analysis of relative mRNA expression of IL-12 on DCs in each group (n = 3). **l** CD4^+^ T cells were activated with ConA (5 μg/mL) for 18 h, and then were co-cultured with DCs at a ratio of 1:100. CCK-8 was used to test T cell proliferation (n = 5). Results of experiments were shown as the mean ± SD. Statistical significance was assessed using one-way ANOVA analysis with Sidak’s multiple comparisons test (**i**–**l**) and two-way ANOVA analysis with Sidak’s multiple comparisons test (**b**, **c**, **e**, **f**). *P* values are reported as follows: *, ^#^, ^&^ < 0.05 and **, ^##^, ^&&^ < 0.01

A balance of mitochondrial dynamics is essential for maintaining mitochondrial homeostasis. Mitochondrial dynamics is regulated by the fission protein Drp1, and fusion proteins mitofusin 2 (Mfn2) and optic atrophy 1 (OPA1) (Giacomello et al. 2020). Our previous study has indicated that mitochondrial dynamics shifts toward Drp1-dependent mitochondrial fission in immune cells during sepsis and Mdivi-1, as a selective inhibitor of Drp1, reduced the apoptosis of immune cells through inhibiting mitochondrial fission (Wu et al. 2019). Moreover, an increase in mitochondrial fission contributes to the dysfunction of DC (Zhang et al. 2020). To determine the role of PINK1 in LPS-induced mitochondrial quality control, we secondly evaluated the induction of mitochondrial dynamics in DCs isolated from WT and *PINK1*^*−/−*^ mice following LPS treatment. As shown in Fig. S2c, 6a, b, knockout of *PINK1* reduced both mRNA and protein expression levels of Mfn2 following at 4 h LPS treatment (*P* < 0.01 or *P* < 0.05). Compared with the Wild Type-LPS group, both mRNA and protein expression levels of Drp1 were upregulated following LPS treatment in *PINK1*^*−/−*^-LPS group (Additional file 2: Fig. S2d, 6d, e, *P* < 0.01 or *P* < 0.05). In addition, compared with the Wild Type-LPS group, the average mitochondrial area had non-significant changes following LPS treatment in *PINK1*^*−/−*^-LPS group (Fig. 6f, g, *P* > 0.05). Then, we determined whether the negative role of *PINK1* knockout on DC function was reversed by Mdivi-1. As shown in Fig. 6h–l, Mdivi-1 reversed the negative role of *PINK1* knockout on DC function at 4 h following LPS treatment (*P* < 0.01 or *P* < 0.05).

### Knockout of *PINK1* prevented DC function and increased apoptosis after CLP

**Fig. 7:**
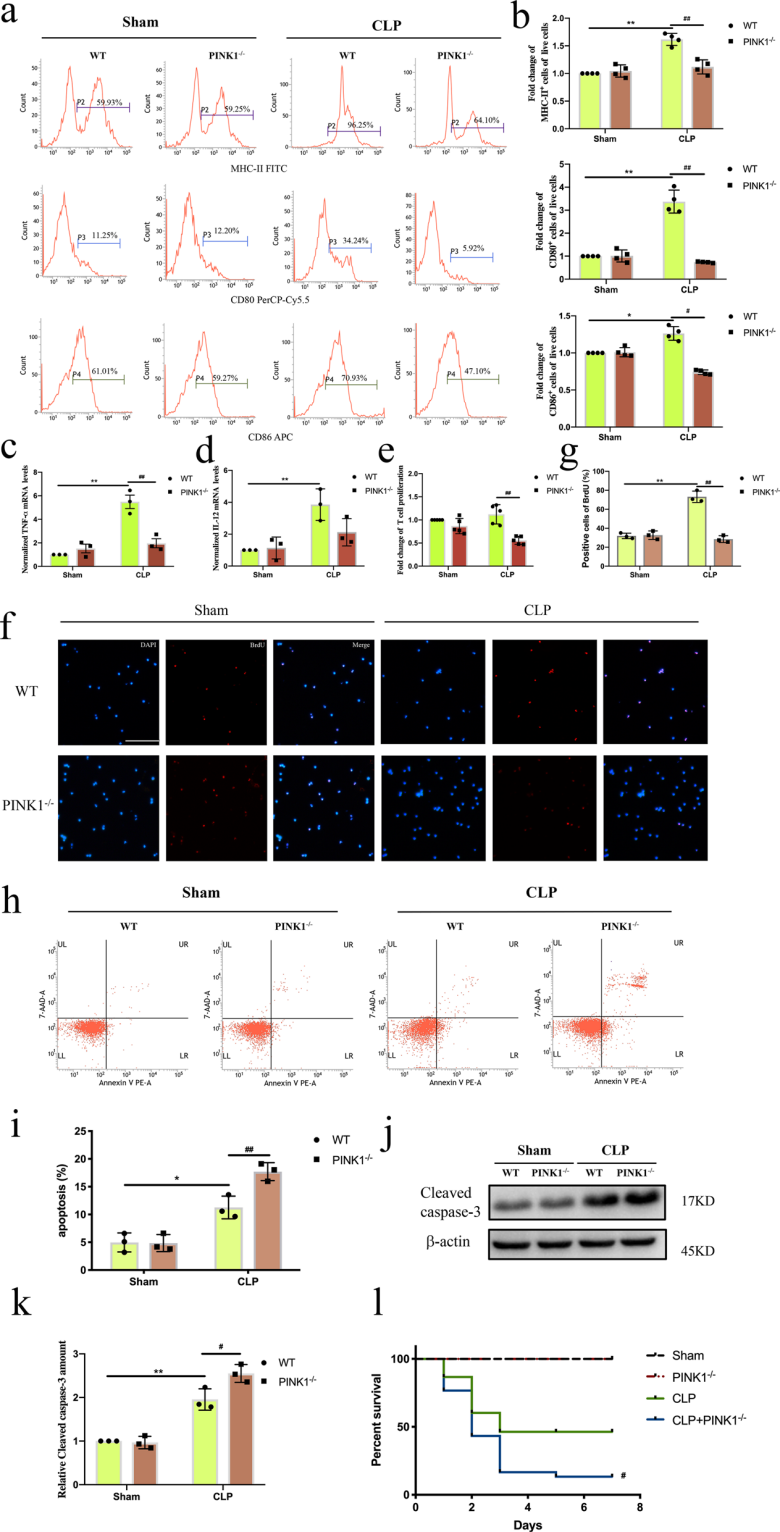
Knockout of *PINK1* prevented DC function and increased apoptosis of DCs after CLP. Splenic DCs were collected from WT mice and *PINK1* knockout mice at 24 h after CLP. **a, b** Representative flow cytometric analysis of MHC-II, CD80, and CD86 expression and statistical analysis of relative MHC-II, CD80, and CD86 expression on DCs in each group (n = 4). **c** TNF-α mRNA expression was analyzed by PCR and statistical analysis of relative mRNA expression of TNF-α on DCs in each group (n = 3). **d** IL-12 mRNA expression was analyzed by PCR and statistical analysis of relative mRNA expression of IL-12 on DCs in each group (n = 3). **e** CCK-8 was used to test T cell proliferation (n = 5). **f, g** Representative images of BrdU staining (red) and DAPI (blue) on DCs in each group and statistical analysis of positive cells (n = 3). **h, i** Apoptosis of DCs was tested by PE-Annexin V and 7-AAD. Representative flow cytometric analysis of apoptosis and statistical analysis of DCs apoptosis in each group (n = 3). **j, k** Representative western blots of cleaved caspase-3 and β-actin in each group and statistical analysis of relative cleaved caspase-3 expression (n = 3). **l** Survival rate of the mice from each group over 7 days. Results of experiments were shown as the mean ± SD. Statistical significance was assessed using two-way ANOVA analysis with Sidak’s multiple comparisons test. *P* values are reported as follows: *, ^#^ < 0.05 and **, ^##^ < 0.01

Finally, we examined whether PINK1 affected the function, apoptosis, and mortality of DCs after CLP. For instance, we evaluated phenotypic maturation and cytokine expression of DCs isolated from WT and *PINK1*^*−/−*^ mice subjected to CLP. Flow cytometry analysis showed that the ratio of DCs expressing MHC-II, CD80, and CD86 after CLP was decreased in *PINK1*^*−/−*^ mice compared to that in WT mice (Fig. 7a, b, *P* < 0.01 or *P* < 0.05). Furthermore, compared with those from WT mice, the mRNA expression of TNF-α and IL-12 was decreased in DCs from *PINK1*^*−/−*^ mice at 24 h after CLP (Fig. 7c, d, *P* < 0.01). Consistent with these findings, DC-mediated T-cell proliferation was also decreased in *PINK1*^*−/−*^ mice compared to that in WT mice at 24 h post CLP (Fig. 7e–g, *P* < 0.01). Furthermore, we examined apoptosis of DCs isolated from WT and *PINK1*^*−/−*^ mice subjected to CLP, as well as the mortality of the mice. As shown in Fig. 7h, i, the level of DC apoptosis was upregulated in *PINK1*^*−/−*^ mice compared to that in WT mice after CLP (*P* < 0.01 or *P* < 0.05). In the western blot assay, knockout of *PINK1* increased cleaved caspase-3 expression (Fig. 7j, k, *P* < 0.01 or *P* < 0.05). The survival rate was also decreased for *PINK1*^*−/−*^ mice after CLP compared to that in WT mice (Fig. 7l, *P* < 0.05). These results support the notion that knockout of *PINK1* prevented DC function and increased DC apoptosis.

## Discussion

It has been increasingly revealed that most individuals are able to initially overcome hyper-inflammatory responses, but may rapidly die days or weeks later. Recent studies have verified that the high mortality of patients in the late phase of sepsis is associated with immunosuppression. The state of immunosuppression renders the individual incapable of resisting their primary infection and increases the possibility of secondary infection. Thus, understanding the pathophysiology of immunosuppression and identifying specific interventions for reversing immunosuppression may allow for improved outcomes for patients with sepsis (Stolk et al. 2020).

DCs are the most important APCs in immune responses and function as a bridge between innate immunity and adaptive immunity. Emerging evidence suggests that DC dysfunction plays an important role in immunosuppression caused by cancer, infection, and sepsis (Wu et al. 2017). During sepsis, DC function is followed by functional changes, including their migration to lymph nodes, the secretion of pro-inflammatory cytokines, and activation of naive T cells in the initial stage; however, DC function is thereafter impaired, cytokines secretion is decreased, and DC dysfunction leads to T-cell anergy in the late stage (Bouras et al. 2018). It is suggested that the function of DCs is gradually impaired during the progression of sepsis, which has been proposed to be the underlying etiology of the associated immunosuppression. In our study, we firstly verified whether the function of DCs was changed during sepsis. We found that the ratio of DCs expressing MHC-II, CD80, and CD86, the mRNA expression levels of TNF-α and IL-12 by DCs, and the level of DCs-mediated T-cell proliferation were all increased after CLP, peaked at 24 h. Consistent with these results, the ratio of DCs expressing MHC-II, CD80, and CD86, the mRNA expression levels of TNF-α and IL-12 by DCs, and the level of DCs-mediated T-cell proliferation also increased following LPS treatment, peaked at 4 h. Our results are also consistent with previous studies that report the function of DCs, especially mature DCs, is impaired after 24 h of CLP. We therefore further investigated the potential factors that lead to the change in DC function during sepsis.

Our previous research found that the imbalance of mitochondrial dynamics leads to increased mitochondrial fragmentation, which was responsible for T cell apoptosis under sepsis (Wu et al. 2019). Notably, recent studies have demonstrated that collective mitochondrial fragmentation is involved in the immune tolerance and apoptosis of immune cells (Mukherjee et al. 2022; Zheng et al. 2019). Recent research has indicated that an increase in mitochondrial fragmentation contributes to the dysfunction of DCs (Wculek et al. 2019; Zhang et al. 2020). Moreover, specific interventions eliminate collective mitochondria fragmentation will ameliorate DC dysfunction. How is mitochondrial fragmentation regulated? The mitochondrial serine/threonine kinase PINK1 has been characterized as a guide for damaged mitochondria. It is widely accepted that PINK1 deficiency is the cause of familial recessive Parkinsonism. Moreover, PINK1 deficiency contributes to the severity of multiple organ injury during sepsis and increases the mortality rate of CLP mice (Kang et al. 2016). Interestingly, Matheoud and colleagues found that PINK1 or Parkin deficiency reduces mitochondrial antigen presentation in macrophage cells, which relies on the generation of mitochondrial derived vesicles (Matheoud et al. 2016). Nevertheless, changes in PINK1 expression of DCs during sepsis and the effect of PINK1 on DC function are largely unknown. We found that the expression levels of mitochondrial PINK1 in DCs were increased after CLP, peaked at 24 h. Furthermore, the expression levels of mitochondrial PINK1 in DCs also increased following LPS treatment, peaked at 4 h. Interestingly, these results suggest that the changes in mitochondrial PINK1 expression of DCs parallel the changes in DC function after CLP. Collectively, we hypothesize that the changes in DC function may be related to the changes in mitochondrial PINK1 expression during sepsis.

Next, we examined whether PINK1 affected DC function. It is well known that the function of DCs is characterized by a mature DC phenotype, including expression of MHC-II, CD80, and CD86, the production of pro-inflammatory cytokines, and the induction of T-cells proliferation. Of note, we found that the ratio of DCs expressing MHC-II, CD80, and CD86 expression, the level of mRNA expression of TNF-α and IL-12 by DCs, and the level of DCs-mediated T-cell proliferation was all decreased in *PINK1*^*−/−*^ mice compared to that in WT mice after CLP. It was noticed in in vitro experiments that DCs with *PINK1* knockout exhibited reduced percentages of cells expressing MHC-II, CD80, and CD86, reduced levels of mRNA expression of TNF-α and IL-12, and reduced DCs-mediated T-cell proliferation following LPS treatment. Surprisingly, the above noted results indicate that *PINK1* knockout prevented DC function. Therefore, the underlying mechanism requires further investigation.

What is the potential mechanism of the negative effect of *PINK1* knockout on DC function? Growing evidence showed when PINK1 detects healthy mitochondria, it is translocated to the OMM and is inserted into the IMM where the matrix processing peptidase (MPP) recognizes and cleaves PINK1, which in turn is released to the cytosol (Murakami et al. 2021). However, when PINK1 detects damaged mitochondria, its import to the IMM is blocked, resulting in the accumulation of PINK1 on the OMM (Tang et al. 2021). PINK1 that is activated through auto-phosphorylation can phosphorylate ubiquitin, a substrate of PINK1, which induces the recruitment of Parkin to the damaged mitochondria. Thereafter, Parkin is activated by phosphorylation and binds to the OMM proteins and autophagy adaptor proteins, including OPTN and NDP52. Then, OPTN and NDP52 bind to LC3II, ultimately leading to mitophagy. We hypothesize that the negative effect of *PINK1* knockout on DC function may be through inhibiting mitophagy. In our data, the expression of PARK2 and LC3 II were increased following LPS treatment and peaked at 4 h, whereas the expression levels of OMM protein Tomm20 were reduced and reached their lowest level at 4 h, suggesting that mitophagy was induced in the initial stage of sepsis and reduced in the late stage of sepsis. Knockout of *PINK1* inhibited LPS-induced upregulation of Parkin and LC3 II and downregulation of Tomm20, suggesting that knockout of *PINK1* inhibited LPS-induced mitophagy. Additionally, overexpression of Parkin reversed the negative role of *PINK1* knockout on DC function at 4 h following LPS treatment. These results support the notion that PINK1 protected against DC dysfunction during sepsis by inducing mitophagy. Collective mitochondrial fragmentation is due to impaired degradation and increased generation. The above data show that the negative effect of *PINK1* knockout on DC function is through inhibiting mitophagy. It would be interesting to know whether *PINK1* knockout induced increased generation of mitochondrial fragmentation? Recent studies have shown that activated PINK1 is involved in regulating mitochondrial dynamics (McLelland et al. 2014; Truban et al. 2017). A balance of mitochondrial dynamics is essential for maintaining mitochondrial homeostasis. Mitochondrial dynamics is regulated by the fission protein Drp1 and its receptors mitochondrial fission factor (Mff), and mitochondrial outer membrane protein Mfn2 and mitochondrial inner membrane protein OPA1 (Giacomello et al. 2020). During mitochondrial fission, Drp1 is transported from the cytosol to the OMM and forms oligomeric Drp1 complexes. Drp1 wraps and constricts the mitochondrial tubule, dissecting the parent mitochondria into two daughters. During mitochondrial fusion, Mfn2 forms homo-oligomeric structures to link two neighboring mitochondria for fusion. In pathological conditions, mitochondrial dynamics shifts toward Drp1-dependent mitochondrial fission that induces the generation of mitochondrial fragmentation. In our data, knockout of *PINK1* reduced Mfn2 levels and upregulated Drp1 levels following 4 h LPS treatment. However, *PINK1* knockout did not affect OPA1 levels. When mitochondria are damaged, PINK1 accumulates in the OMM to clear the damaged mitochondria by regulating mitochondrial quality control through the induction and phosphorylation of OMM proteins, including Mfn2, Mfn1, and VDAC. OPA1 is a mitochondrial inner membrane protein, and therefore, PINK1 does not affect OPA1 levels. Furthermore, we found that Mdivi-1, as a selective inhibitor of Drp1, reversed the negative effect of PINK1 deficiency on DC function. These results support the concept that the negative effect of PINK1 deficiency in DC function may be also associated with an increase in the generation of mitochondrial fragmentation. Mitophagy and mitochondrial dynamics are the processes of mitochondrial quality control, and therefore, we conclude that PINK1 protected against DC dysfunction during sepsis through the regulation of mitochondrial quality control.

Finally, we determined the effect of PINK1 on DC apoptosis and mortality during sepsis. We revealed that DC apoptosis and the mortality rate increased in *PINK1*^*−/−*^ mice after CLP compared with that in WT mice. In summary, results from the current study support the concept that the changes in mitochondrial PINK1 expression of DCs during sepsis parallel the changes in DC function. Furthermore, the percentage of DCs expressing MHC-II, CD80, and CD86, the level of mRNA expression of IL-12 and TNF-α by DCs, and the level of DC-mediated T-cell proliferation was all decreased, both in vivo and in vitro, when PINK1 was knocked out, suggesting that *PINK1* knockout prevented the function of DCs during sepsis. Knockout of *PINK1* also increased DC apoptosis and the mortality rate of CLP mice.

Nevertheless, there are several limitations in our study. Firstly, we found the negative effect of PINK1 deficiency on DC function was probably through inhibiting mitochondrial quality control which induced an increase in mitochondrial fragmentation, but the mechanisms of mitochondrial fragmentation causing DC dysfunction need further investigation. Secondly, to strengthen the significance of our study, clinical studies need approved.

**Fig. 8:**
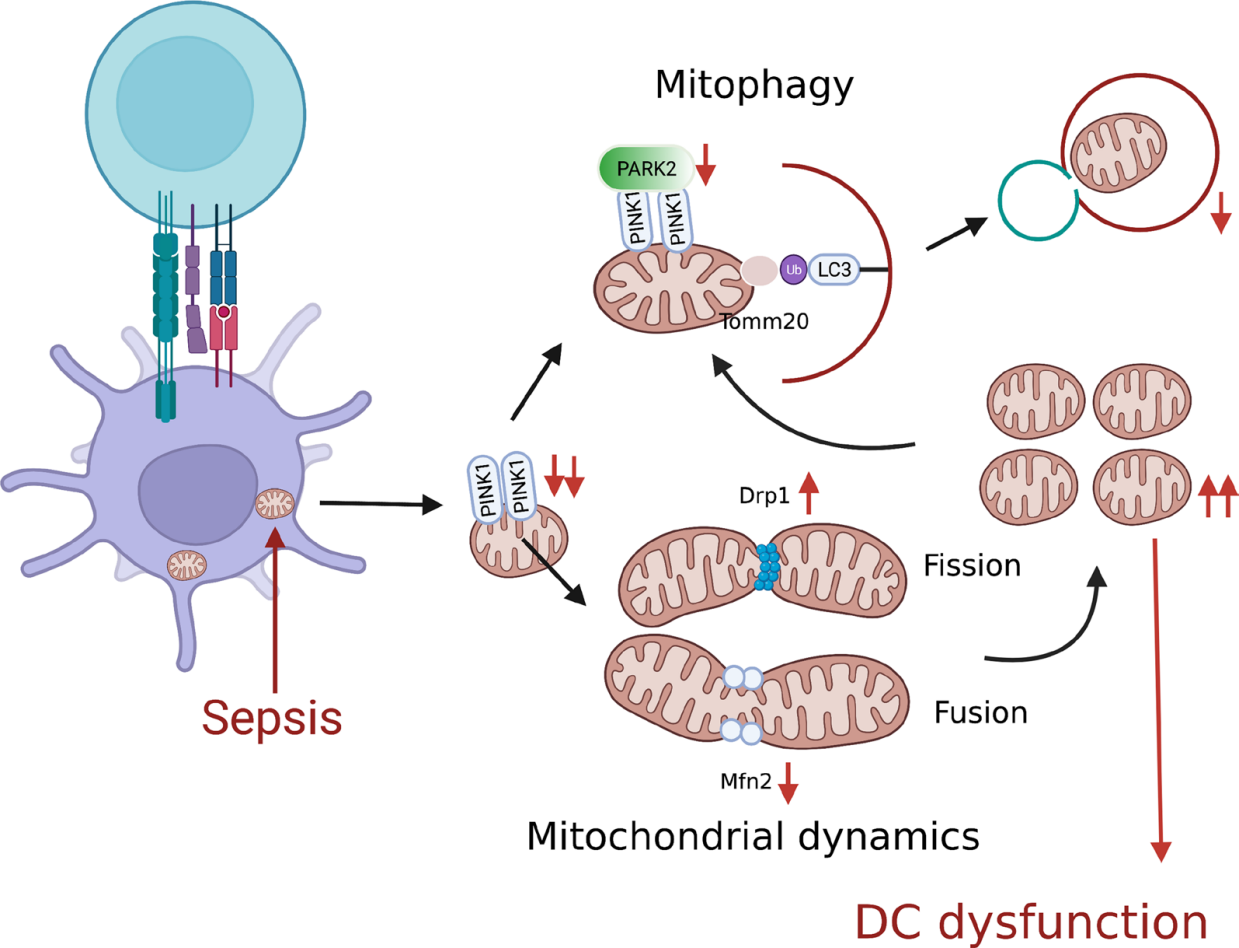
Representative schematic of the hypothesis. Change in mitochondrial PINK1 expression of DCs paralleled change in DC function and *PINK1* knockout prevented function of DCs during sepsis. Previous studies have demonstrated that collective mitochondrial fragmentation which is caused by Drp1-dependent mitochondrial fission are involved in the dysfunction and apoptosis of immune cells. PINK1 has been characterized as a guide for damaged mitochondria which eliminates mitochondrial fragmentation through mitochondrial quality control. Therefore, we hypothesize *PINK1* knockout prevented the function of DCs during sepsis through inhibiting mitochondrial quality control

In summary, mitochondrial dynamics shifts toward mitochondrial fission, which provide more ATP to maintain immune function in the initial stage of sepsis. Moreover, mitochondrial quality control capacity is enhanced to keep cell homeostasis by eliminating mitochondrial fragmentation. However, mitochondrial quality control capacity is impaired in the late stage of sepsis and more mitochondrial fragmentation is collected, ultimately leading to DC dysfunction (Fig. 8). This study makes further understand the pathophysiology of immunosuppression and guides to explore specific interventions for reversing immunosuppression and improving outcomes for patients with sepsis.

## Conclusion

Our results indicated that PINK1 protected against DC dysfunction during sepsis through the regulation of mitochondrial quality control, which suggested that PINK1 is a new therapeutic strategy for sepsis.

## Supplementary Information

**Additional file 1.** The mRNA expression levels of PINK1 and LC3-II.

**Additional file 2.** Overexpression of Parkin recovering mitophagy and the mRNA expression levels of Mfn2 and Drp1.

**Additional file 3.** Original gel/blot images.

## Data Availability

The datasets used and/or analyzed during the current study are available from the corresponding author on reasonable request.
