# Supplementary figures and images for: PINK1 protects against dendritic cell dysfunction during sepsis through the regulation of mitochondrial quality control
Source: Mol Med. 2023 Feb 21;29:25. doi: 10.1186/s10020-023-00618-5 (PMC9945621; doi:10.1186/s10020-023-00618-5)

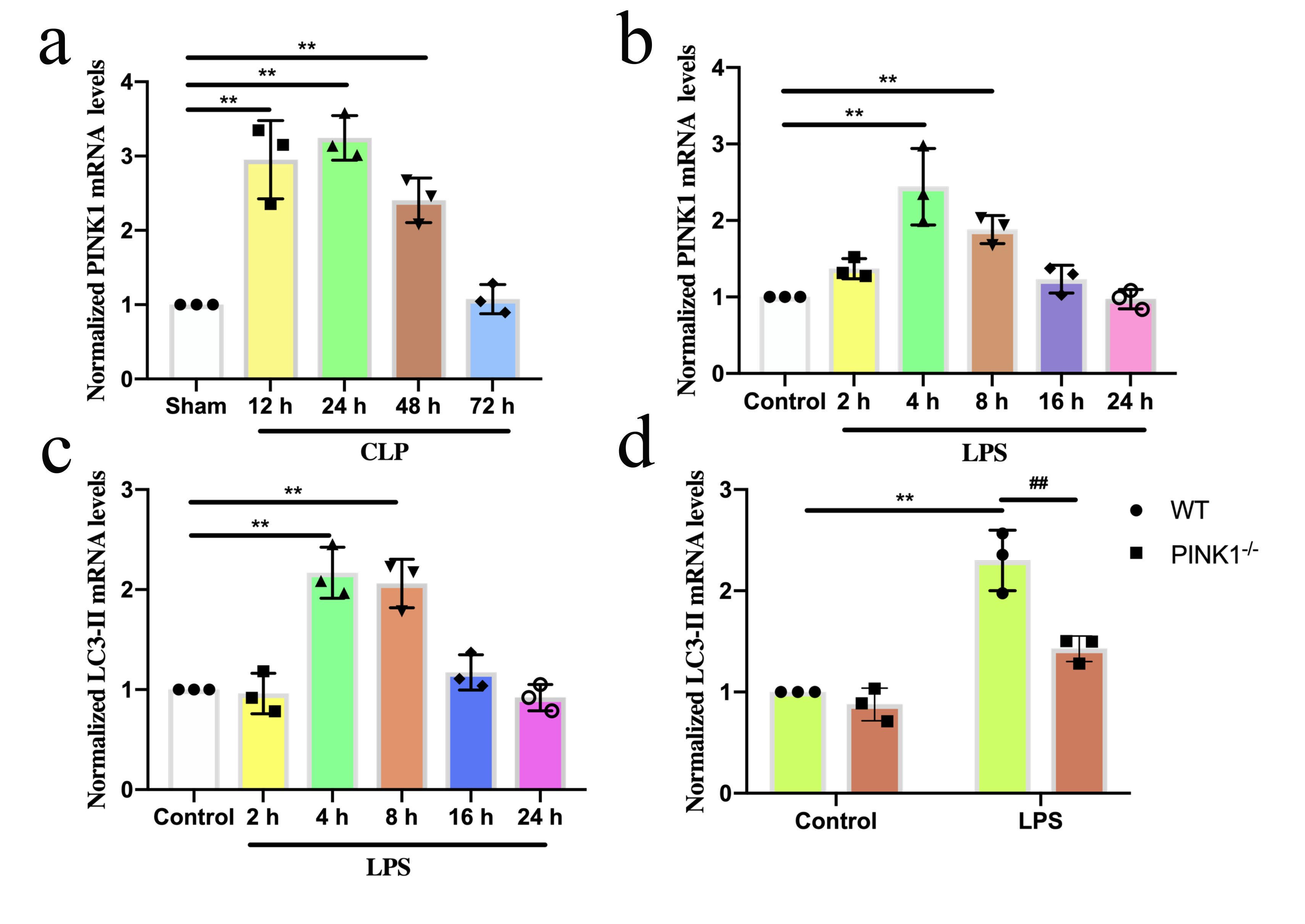

Supplement: Supplementary file 1 — Additional file 1. The mRNA expression levels of PINK1 and LC3-II. [file 10020_2023_618_MOESM1_ESM.png]

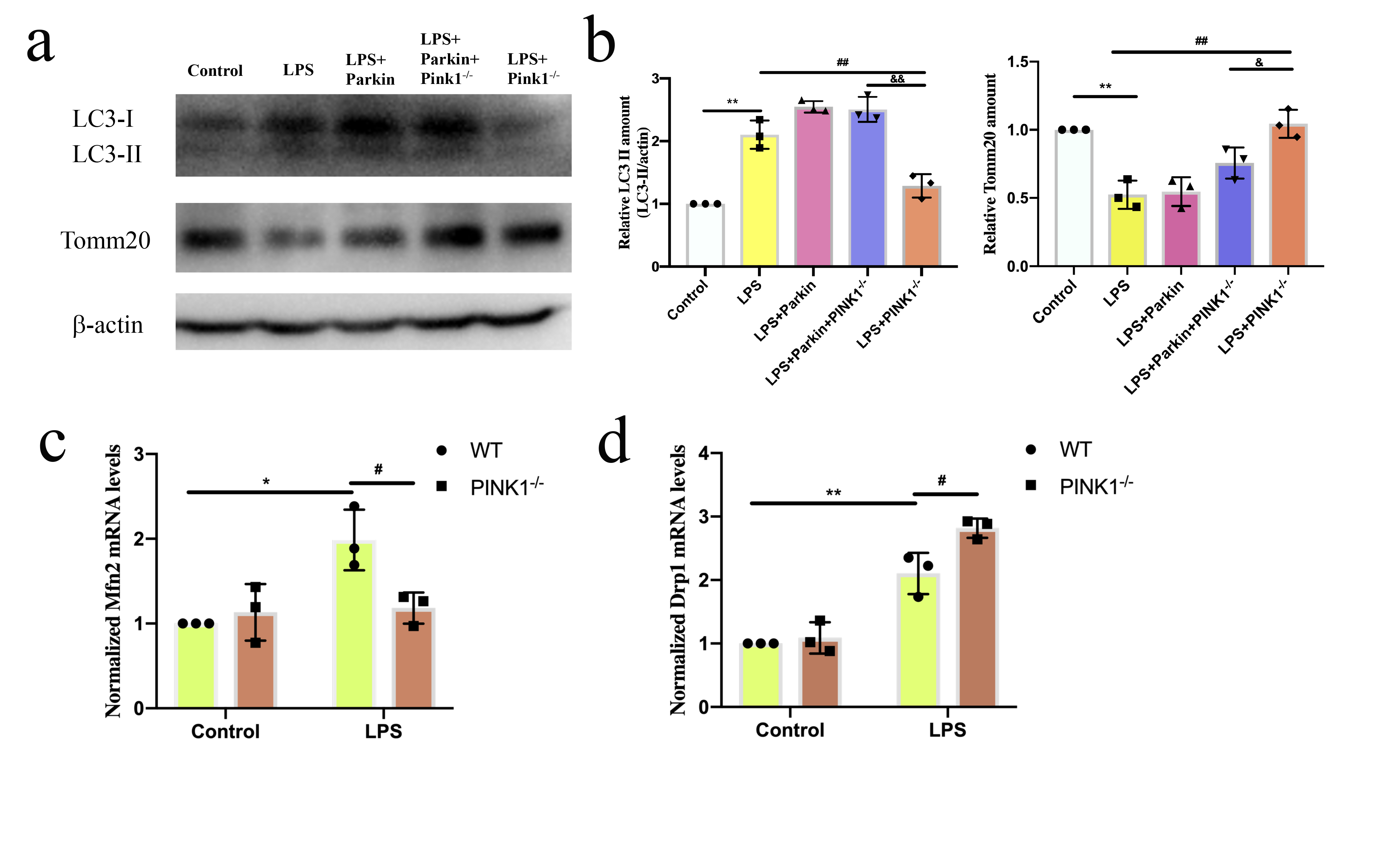

Supplement: Supplementary file 2 — Additional file 2. Overexpression of Parkin recovering mitophagy and the mRNA expression levels of Mfn2 and Drp1. [file 10020_2023_618_MOESM2_ESM.png]
