# Supplementary material for: PINK1 protects against dendritic cell dysfunction during sepsis through the regulation of mitochondrial quality control
Source: Mol Med. 2023 Feb 21;29:25. doi: 10.1186/s10020-023-00618-5 (PMC9945621; doi:10.1186/s10020-023-00618-5)
Supplement: Supplementary file 3 — Additional file 3. Original gel/blot images. [file 10020_2023_618_MOESM3_ESM.pdf]

原始条带-figure 1f

Sham Sham 12 h 24 h 48 h 72 h

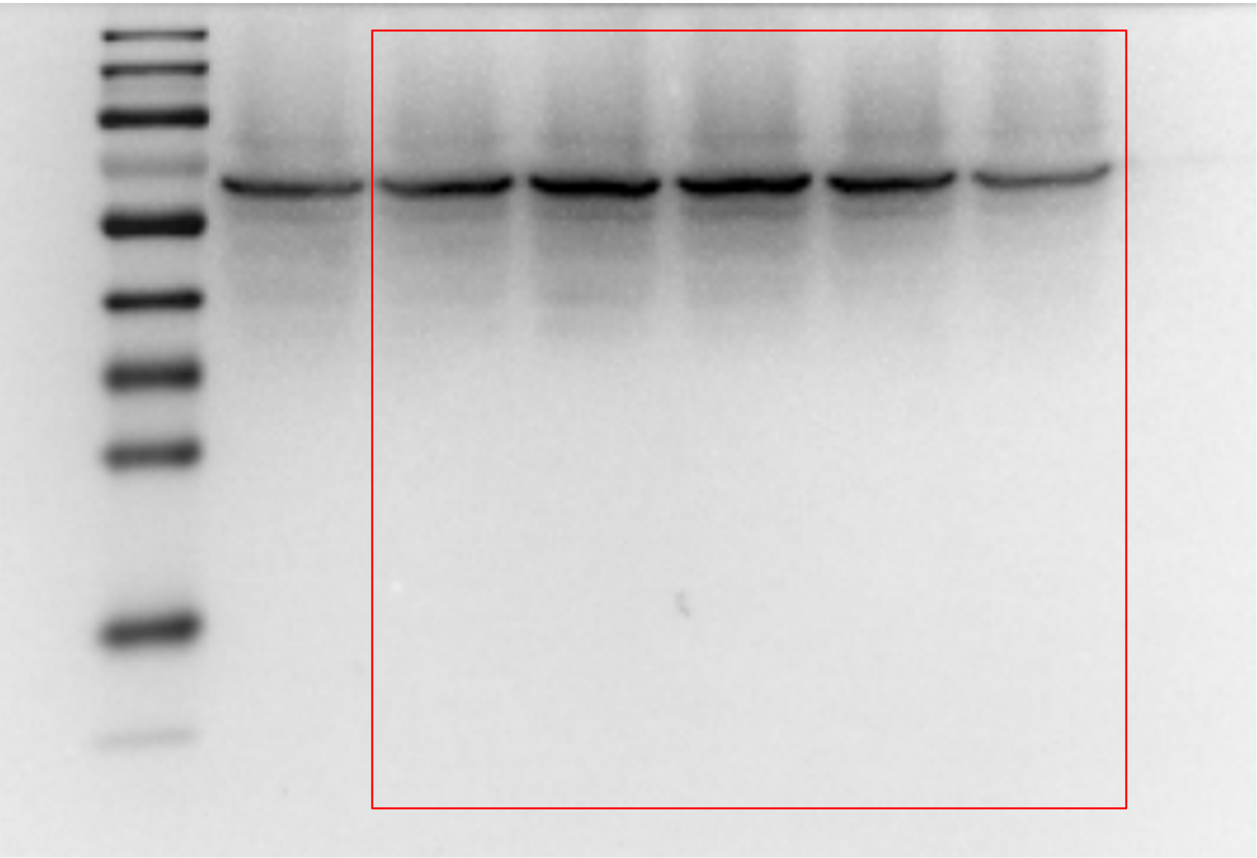

PINK1

Sham Sham 12 h 24 h 48 h 72 h

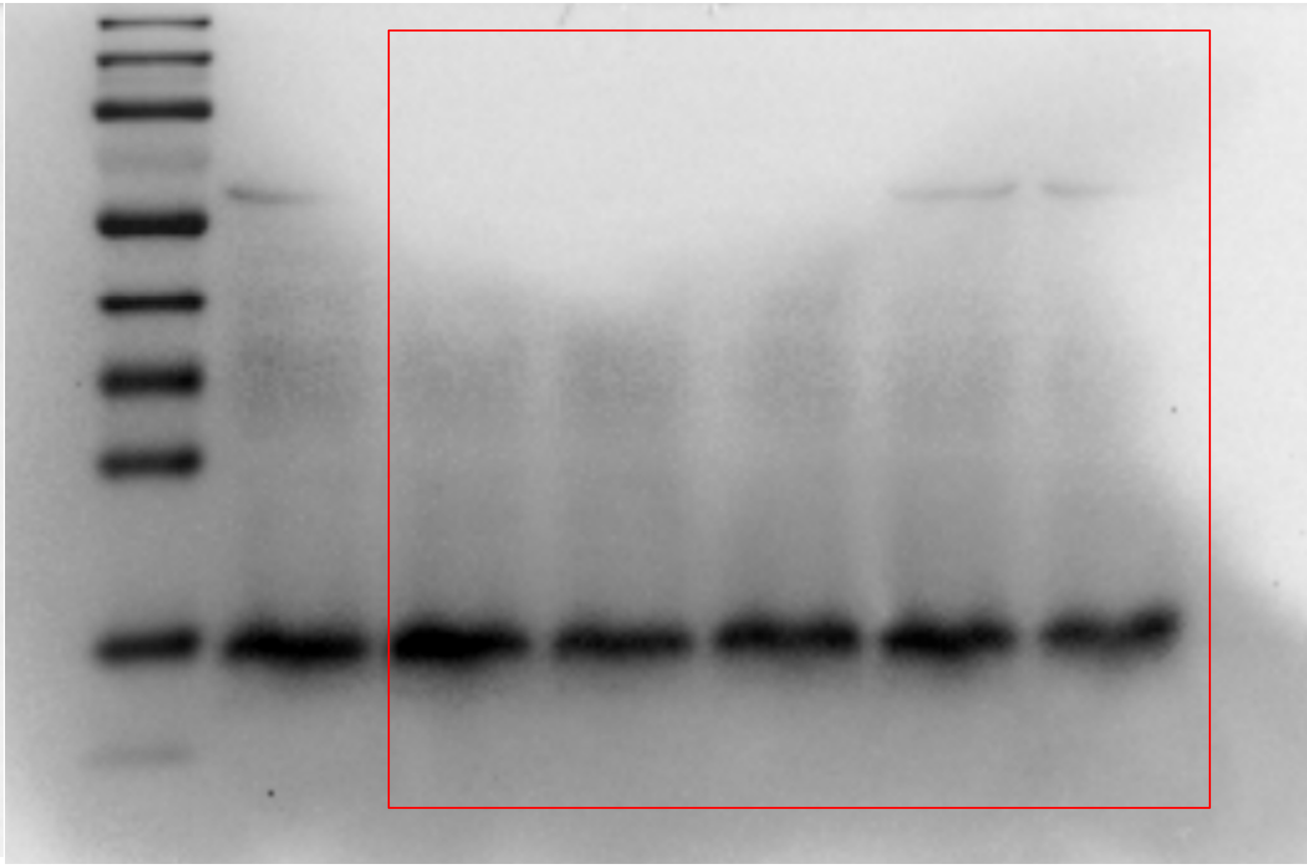

COXIV

原始条带-figure 2f

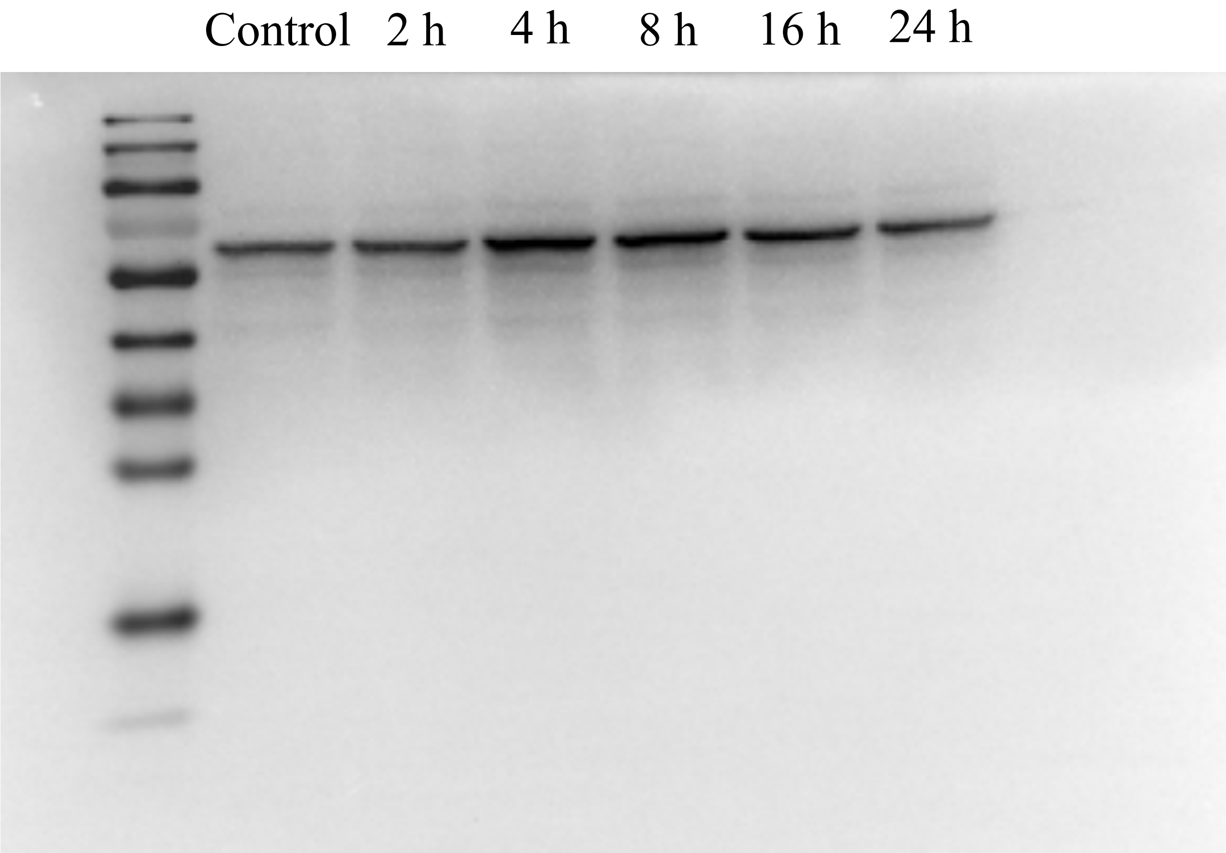

PINK1

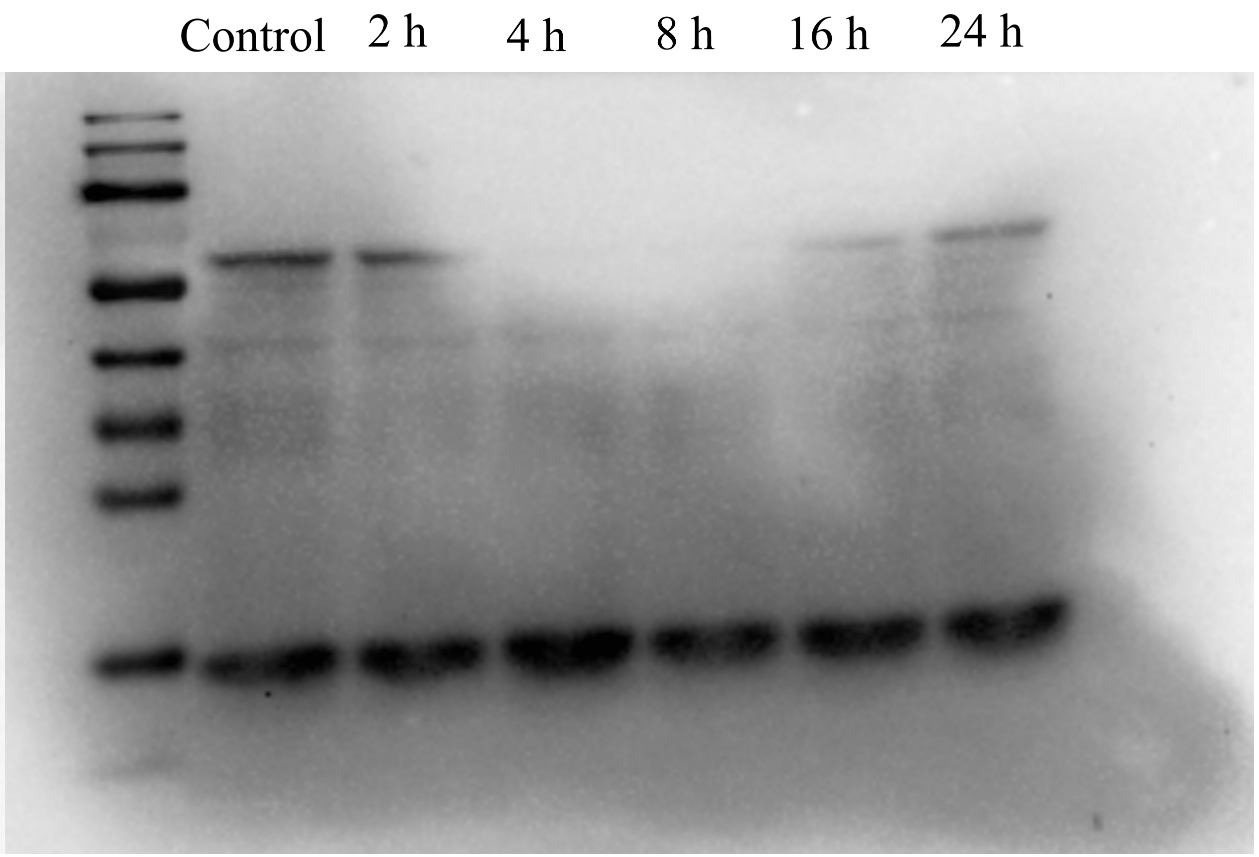

COXIV

原始条带-figure 3a

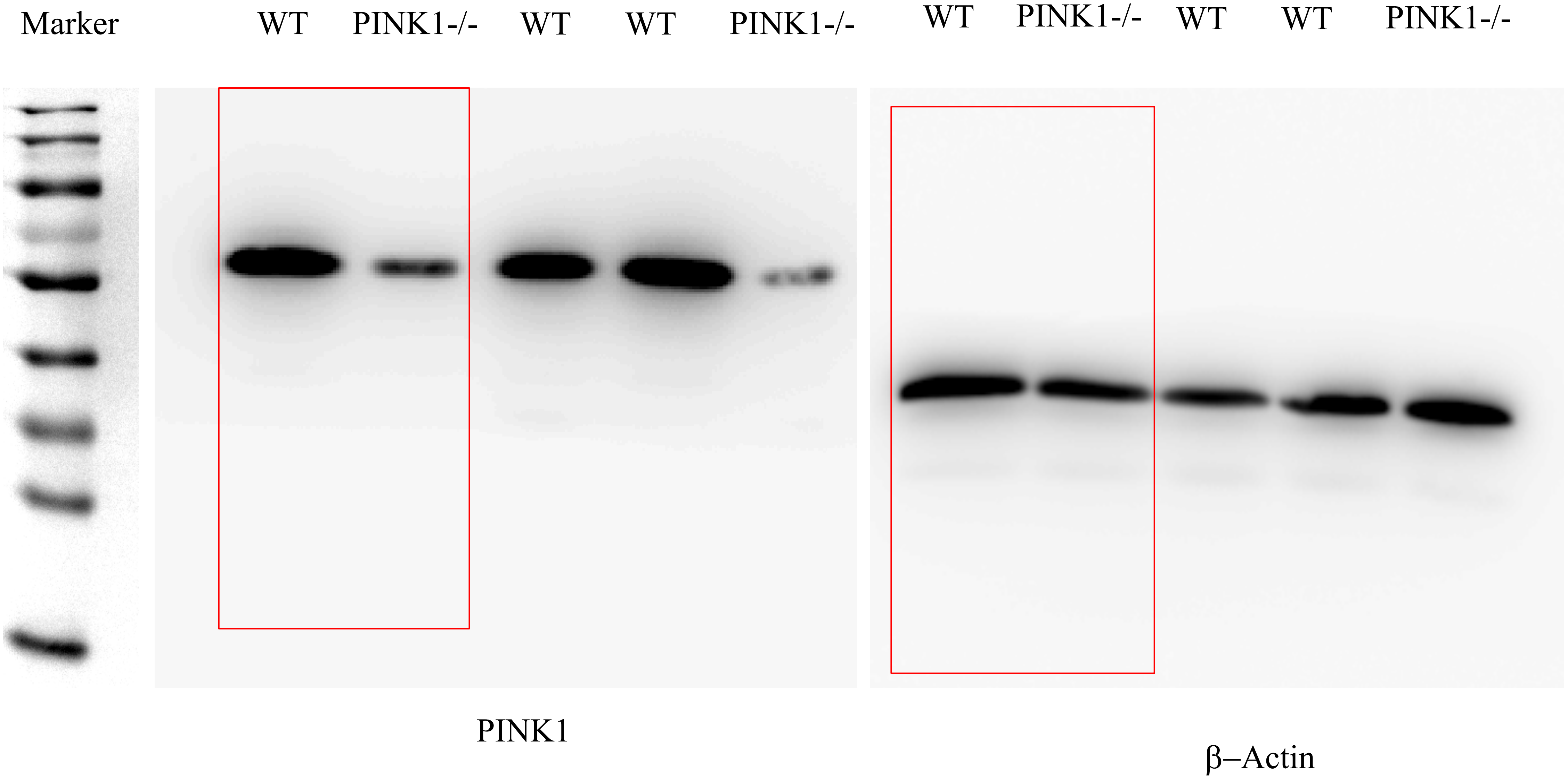

原始条带-figure 4a

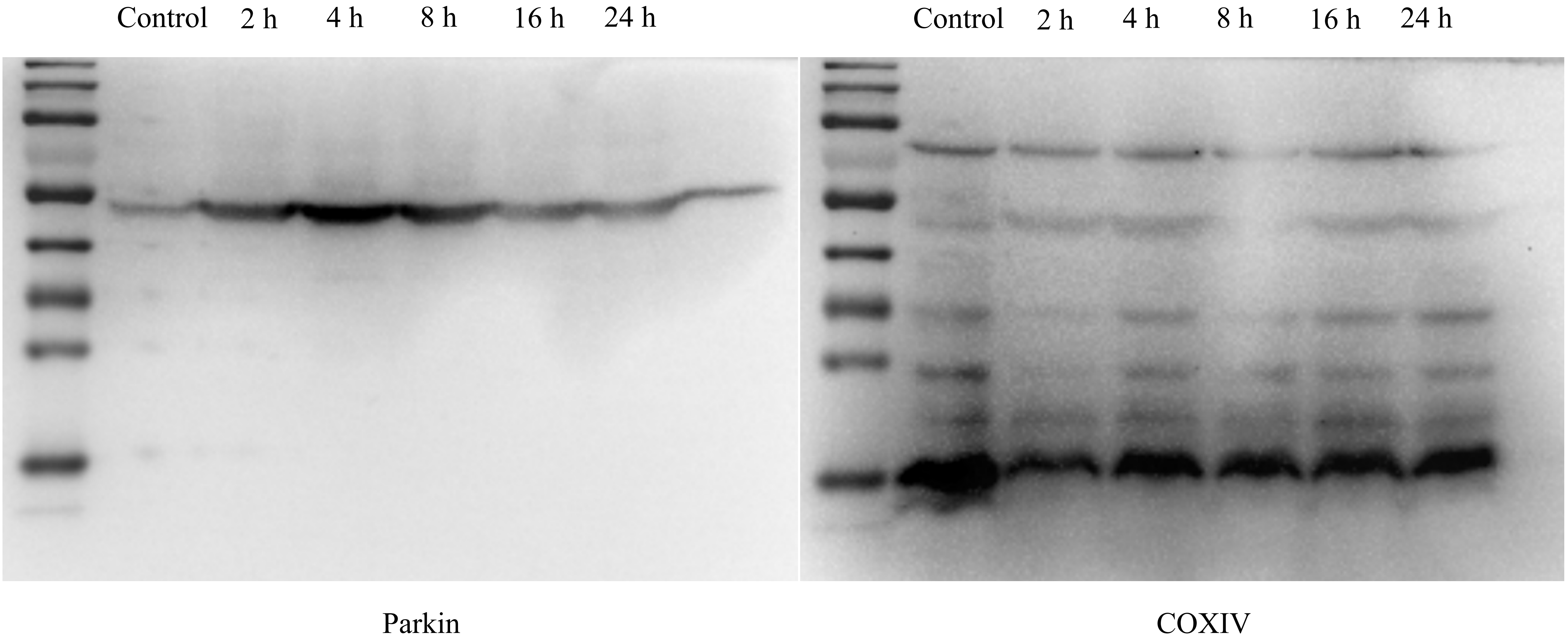

原始条带-figure 4c

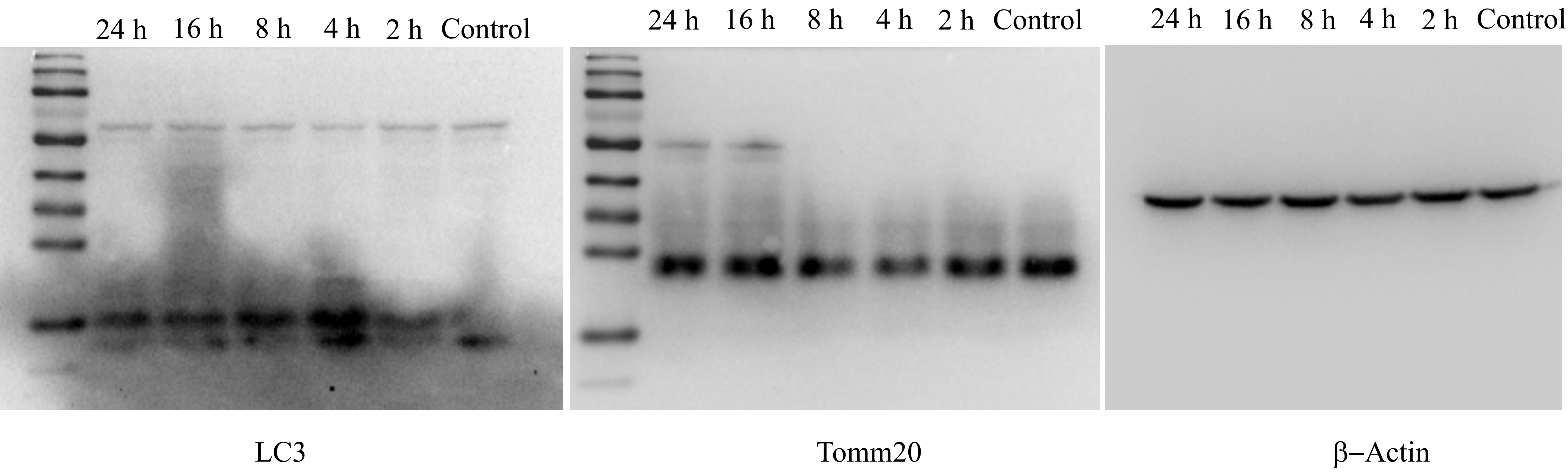

原始条带-figure 5a

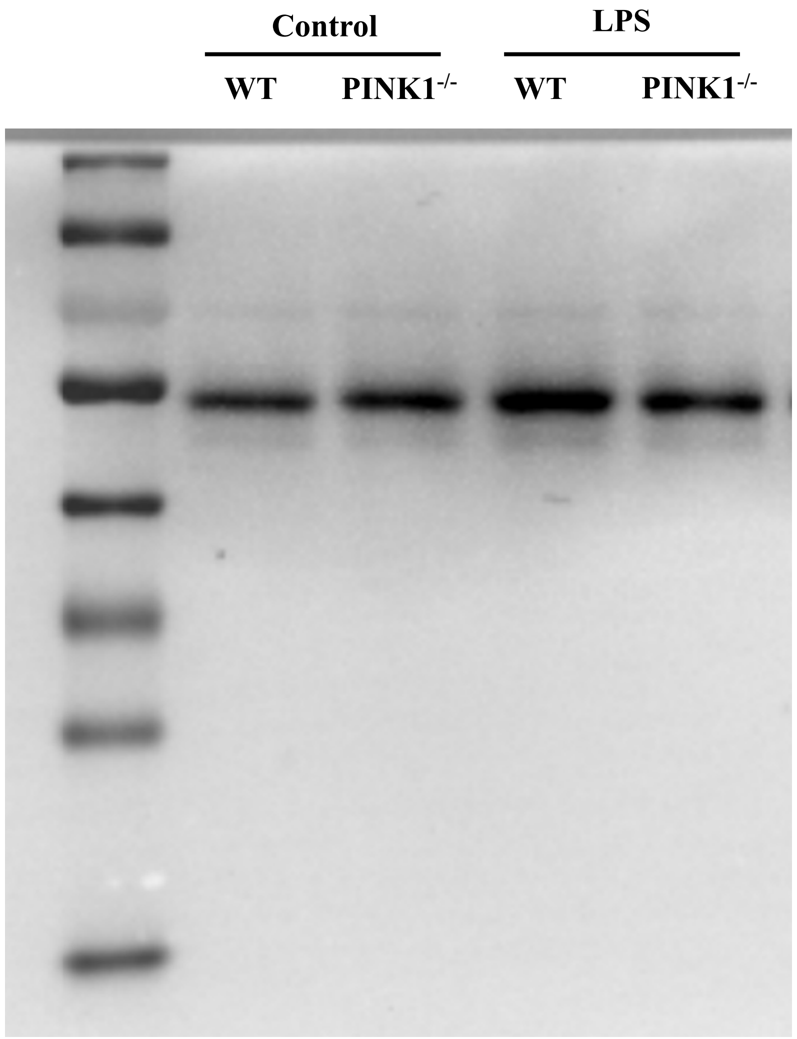

Parkin

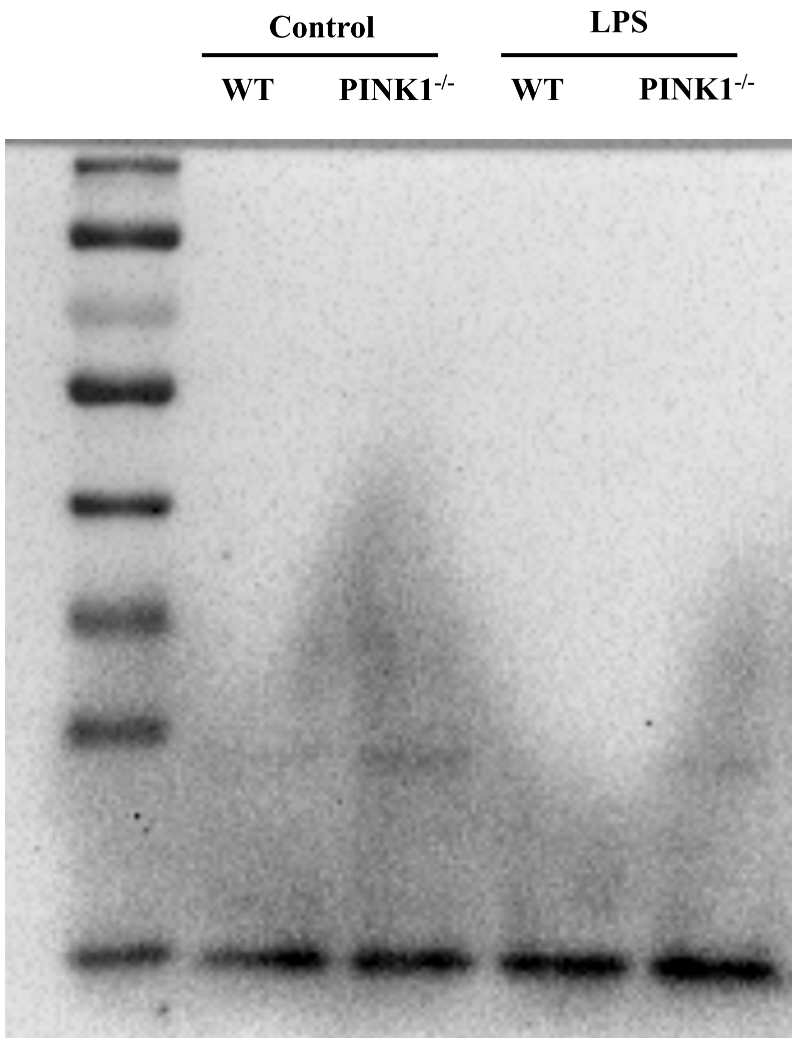

COXIV

原始条带-figure 5d

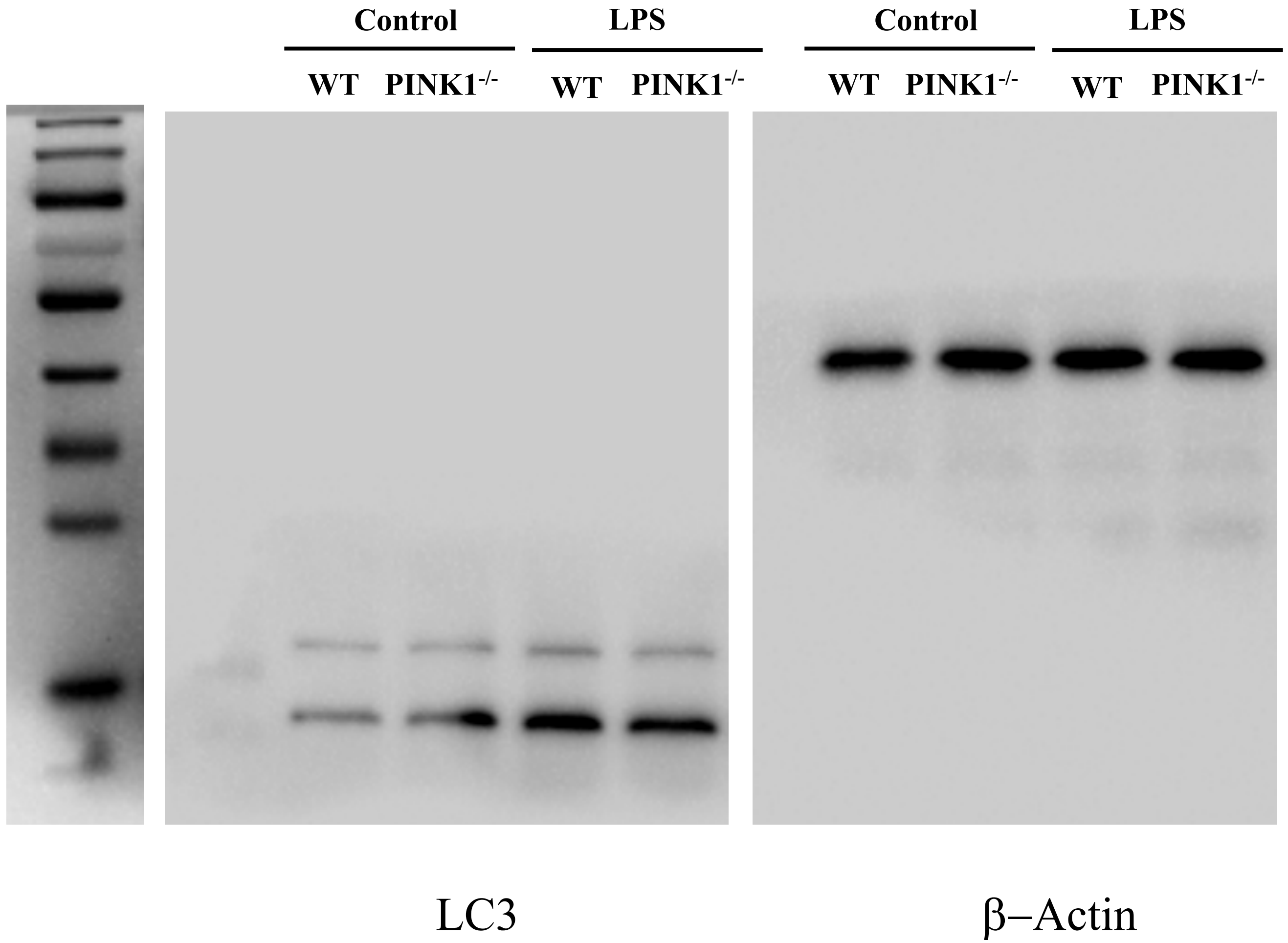

原始条带-figure 5d

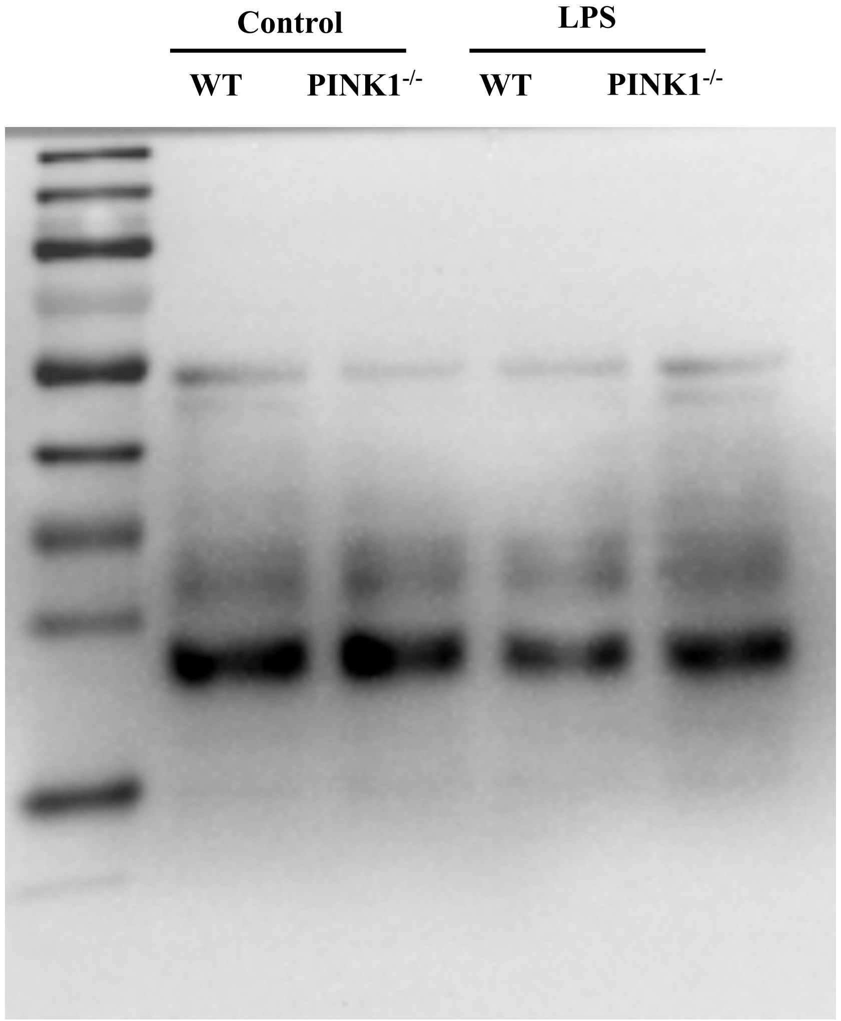

Tomm20

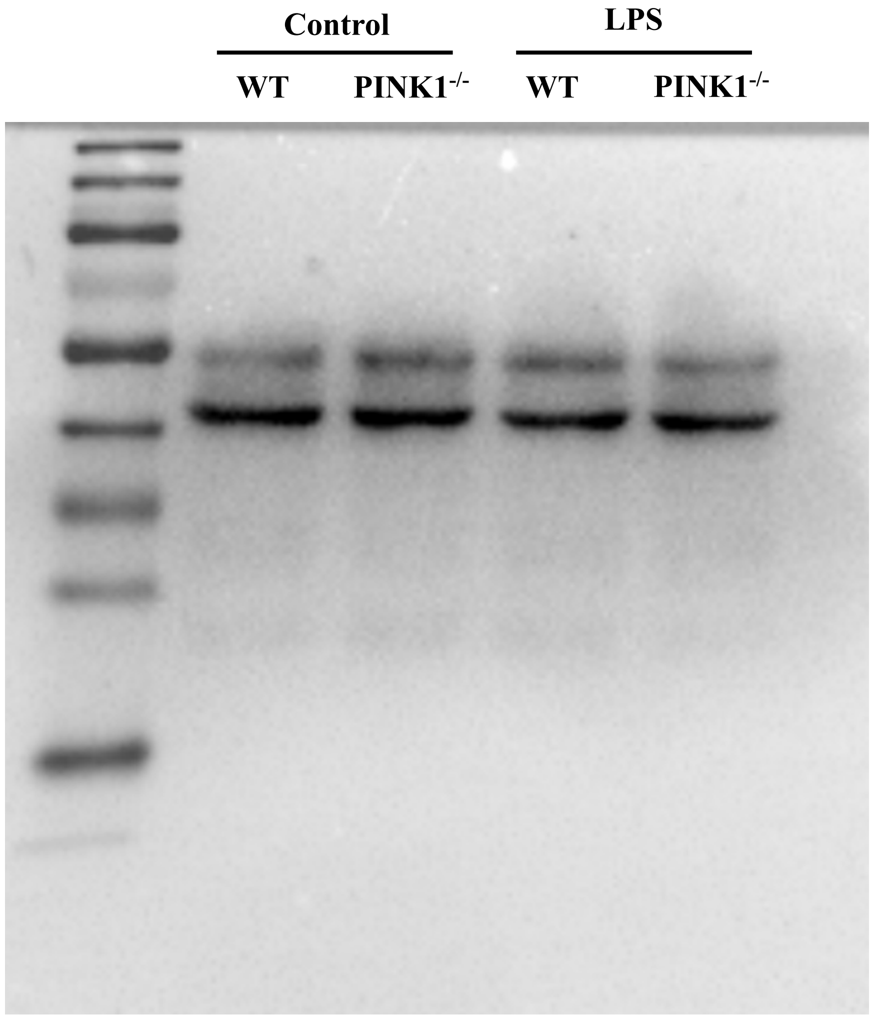

β–Actin

原始条带-figure 6a

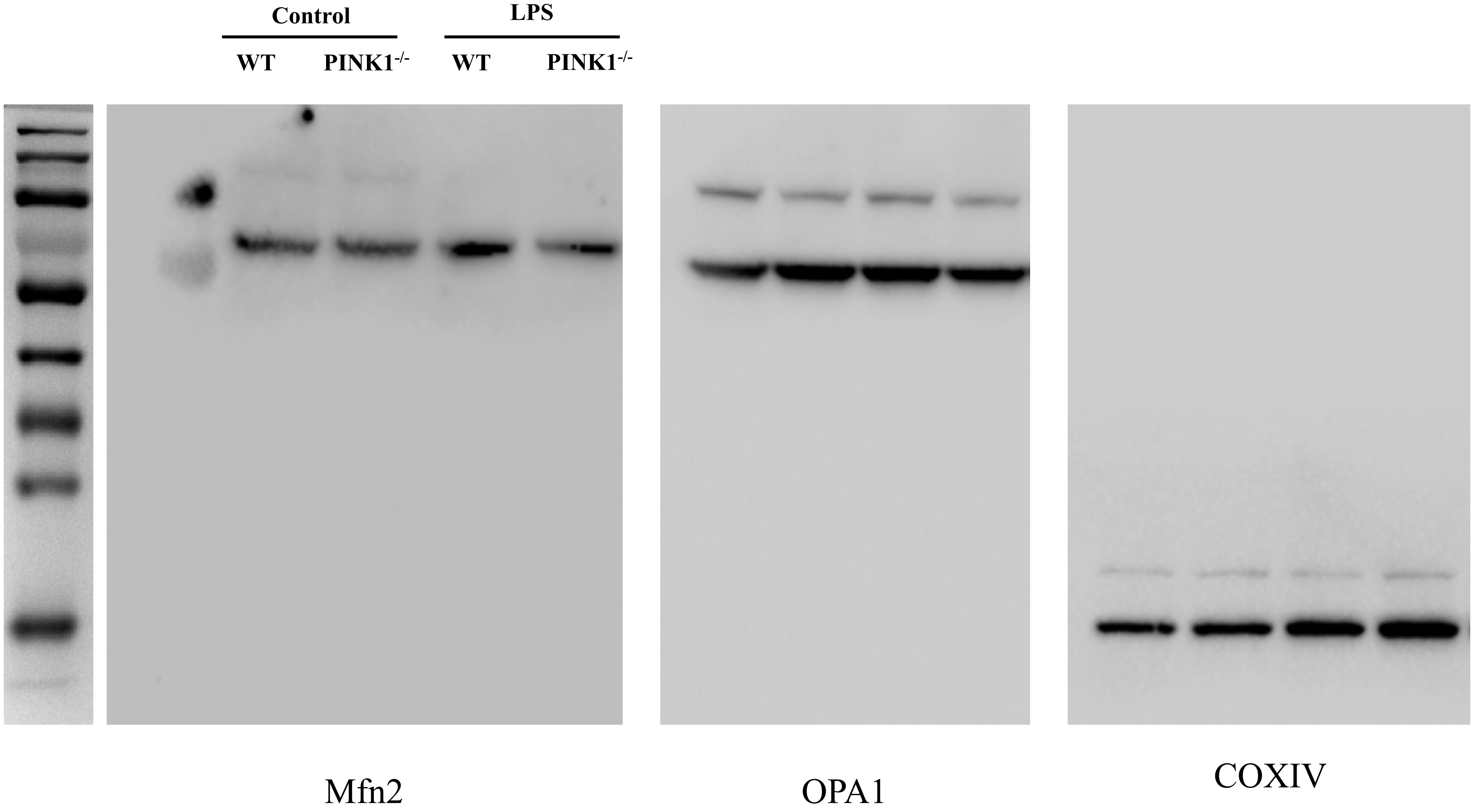

原始条带-figure 6d

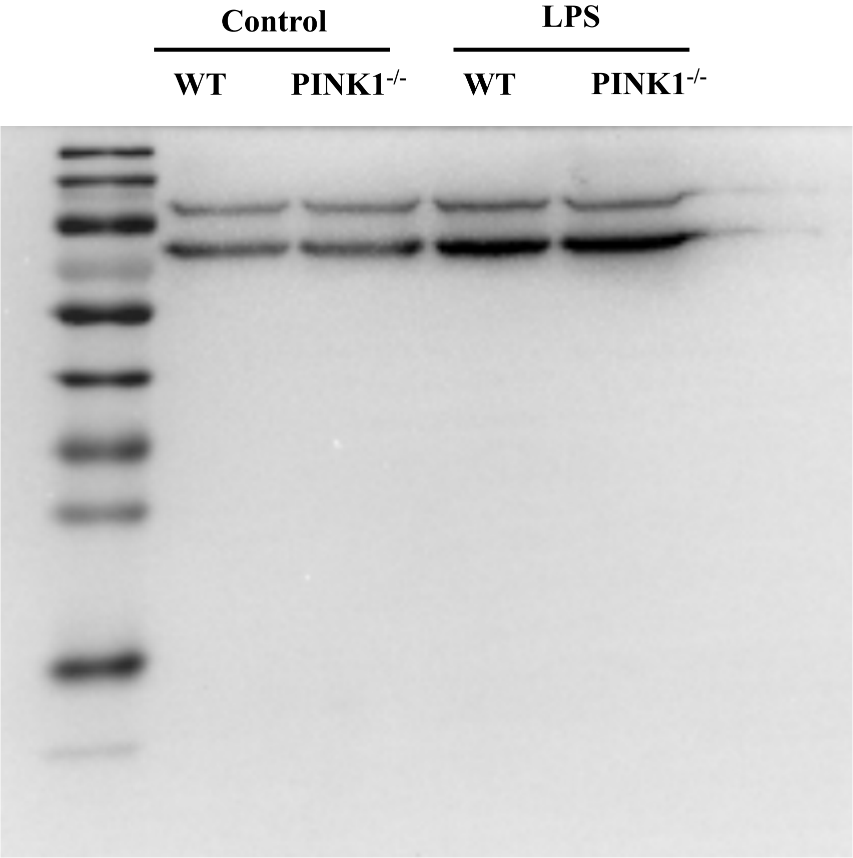

Drp1

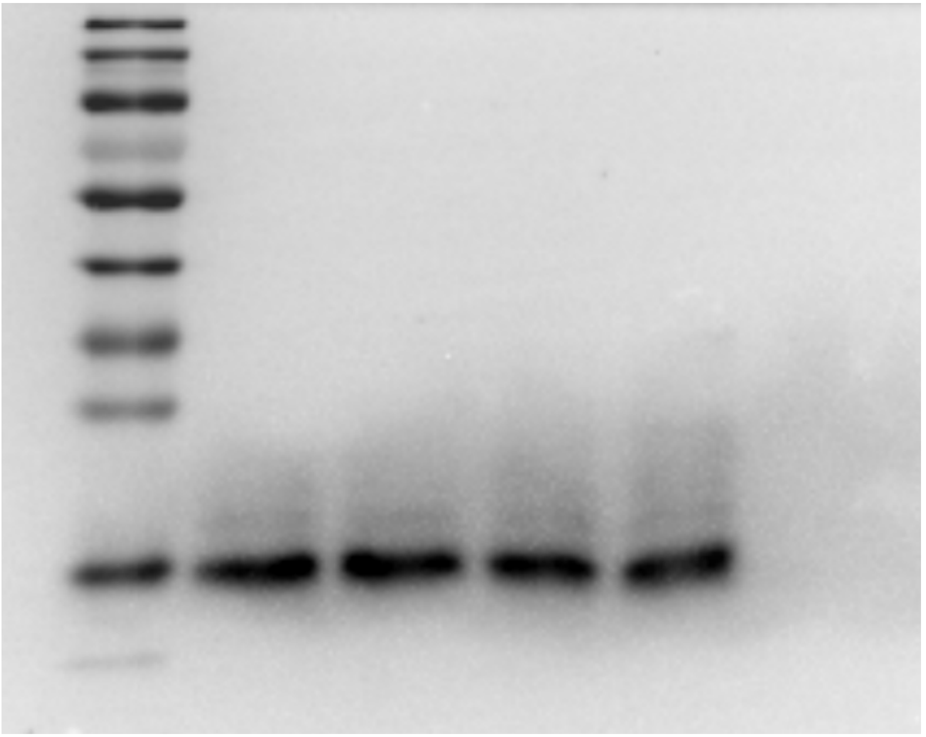

COXIV

原始条带-figure 7j

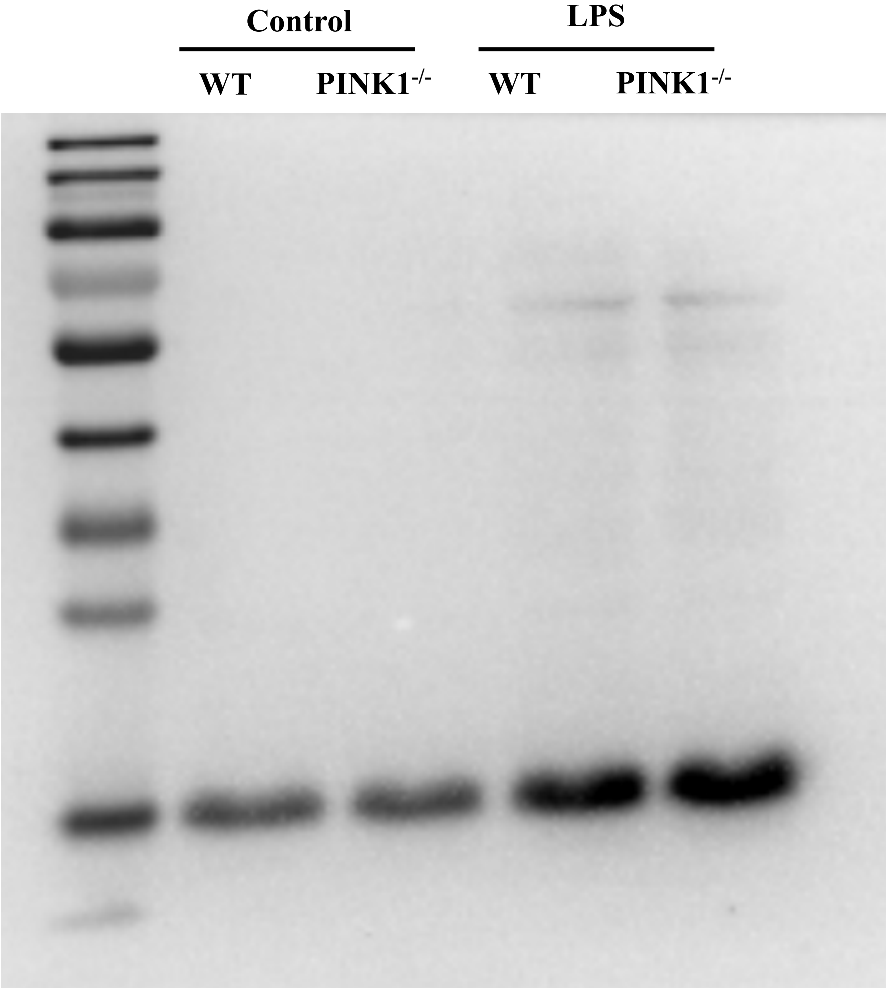

Cleaved caspase-3

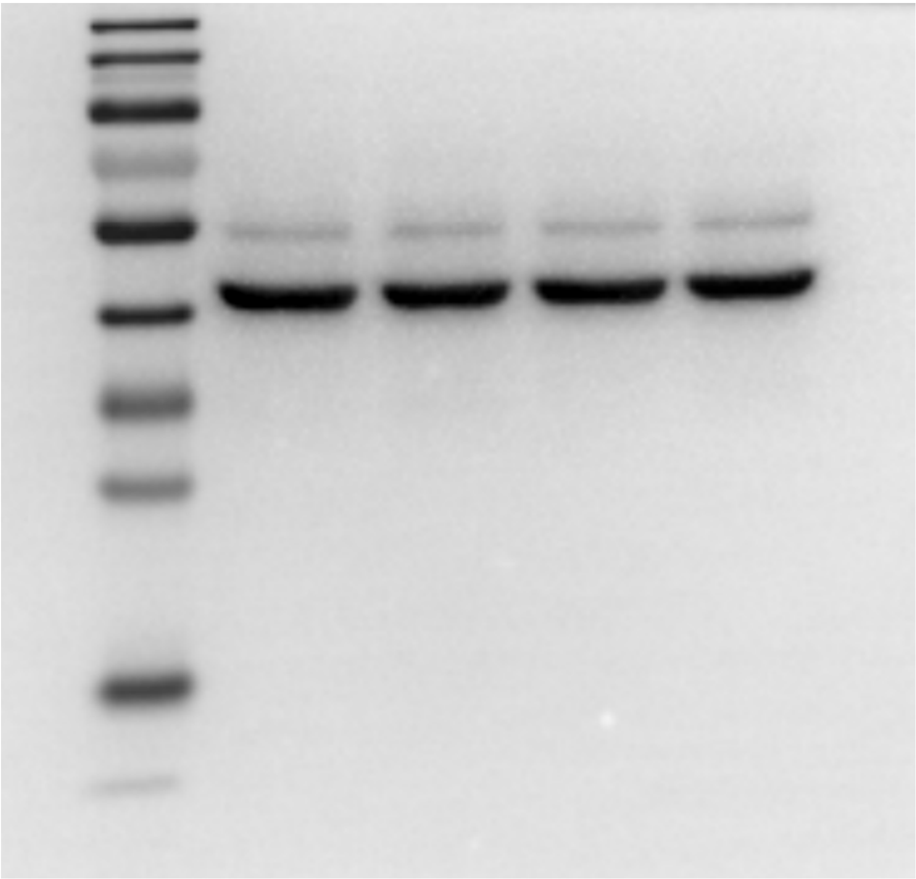

β–Actin
